# Chick Embryo Experimental Platform for Micrometastases Research in a 3D Tissue Engineering Model: Cancer Biology, Drug Development, and Nanotechnology Applications

**DOI:** 10.3390/biomedicines9111578

**Published:** 2021-10-29

**Authors:** Anna Guller, Inga Kuschnerus, Vlada Rozova, Annemarie Nadort, Yin Yao, Zahra Khabir, Alfonso Garcia-Bennett, Liuen (Olivia) Liang, Aleksandra Polikarpova, Yi Qian, Ewa M. Goldys, Andrei V. Zvyagin

**Affiliations:** 1 Faculty of Science and Engineering, Macquarie University, Sydney, NSW 2109, Australia; i.kuschnerus@unsw.edu.au (I.K.); vlada.rozova@hdr.mq.edu.au (V.R.); annemarie.nadort@mq.edu.au (A.N.); zahra.khabir@mq.edu.au (Z.K.); alf.garcia@mq.edu.au (A.G.-B.); liuen.liang@hotmail.com (L.L.); 2 ARC Centre of Excellence for Nanoscale Biophotonics, Macquarie University, Sydney, NSW 2109, Australia; 3 Macquarie Medical School, Macquarie University, Sydney, NSW 2109, Australia; yi.chien.qian@gmail.com; 4 Institute for Regenerative Medicine, Sechenov First Moscow State Medical University (Sechenov University), 119991 Moscow, Russia; 5 ARC Centre of Excellence for Nanoscale Biophotonics, Graduate School of Biomedical Engineering, University of New South Wales, Sydney, NSW 2052, Australia; apolikarpova@yahoo.com; 6 Electron Microscope Unit, Mark Wainwright Analytical Centre, University of New South Wales, Sydney, NSW 2052, Australia; yin.yao@unsw.edu.au; 7 School of Materials Science and Engineering, University of New South Wales, Sydney, NSW 2052, Australia; 8 Institute for Biology and Biomedicine, Lobachevsky State University, 603950 Nizhny Novgorod, Russia; 9 Westmead Hospital, The Westmead Health and Education Super Precinct, Westmead, NSW 2145, Australia; 10 Centre of Biomedical Engineering, Sechenov First Moscow State Medical University (Sechenov University), 119991 Moscow, Russia; 11 Laboratory of NanoOncoTheranostics, Institute of BioOrganic Chemistry, RAS, 117997 Moscow, Russia

**Keywords:** chick embryo, 3D culture, tumor models in vitro, tissue engineering, metastasis, micrometastasis, triple-negative breast cancer, liver, mesoporous silica nanoparticles, doxorubicin

## Abstract

Colonization of distant organs by tumor cells is a critical step of cancer progression. The initial avascular stage of this process (micrometastasis) remains almost inaccessible to study due to the lack of relevant experimental approaches. Herein, we introduce an in vitro/in vivo model of organ-specific micrometastases of triple-negative breast cancer (TNBC) that is fully implemented in a cost-efficient chick embryo (CE) experimental platform. The model was built as three-dimensional (3D) tissue engineering constructs (TECs) combining human MDA-MB-231 cells and decellularized CE organ-specific scaffolds. TNBC cells colonized CE organ-specific scaffolds in 2–3 weeks, forming tissue-like structures. The feasibility of this methodology for basic cancer research, drug development, and nanomedicine was demonstrated on a model of hepatic micrometastasis of TNBC. We revealed that MDA-MB-231 differentially colonize parenchymal and stromal compartments of the liver-specific extracellular matrix (LS-ECM) and become more resistant to the treatment with molecular doxorubicin (Dox) and Dox-loaded mesoporous silica nanoparticles than in monolayer cultures. When grafted on CE chorioallantoic membrane, LS-ECM-based TECs induced angiogenic switch. These findings may have important implications for the diagnosis and treatment of TNBC. The methodology established here is scalable and adaptable for pharmacological testing and cancer biology research of various metastatic and primary tumors.

## 1. Introduction

Metastases remain the leading cause of cancer-related deaths and one of the biggest challenges in oncology [1,2]. During the metastatic cascade, cancer cells shed from the primary tumor and travel through the blood and lymphatic vessels. Circulating tumor cells can escape from the vasculature (extravasation) in a distant organ and attach there (arrest/homing) to form a secondary colony [3]. These colonies are avascular groups of cancer cells, 0.2–2 mm in size, termed micrometastases [4]. If not destroyed by the immune system, micrometastases may either stay dormant or progress to bigger, blood-perfused macrometastases (>2 mm) [4,5,6]. This conversion from micrometastases to massive secondary neoplasms is driven by a tumor-induced acceleration of blood vessel growth in the vicinity of the cancer cell colony (“the angiogenic switch”) [7,8]. 

A fundamental question in cancer biology that is intrinsically coupled with the understanding of the mechanisms of micrometastases is the preferential metastatic organotropism [3,9,10]. This phenomenon of the more frequent formation of metastases of certain types of tumors in certain organs is well-known by Paget’s “seed and soil” metaphor [11]. Notably, the modern vision of the “seeds and soils” principle does not deny the role of the differential blood supply patterns of various organs [12]. In fact, it emphasizes the contribution of the initial interactions between extravasated cancer cells (“the seeds”) and the host microenvironments of distant organs (“the soils”) to the formation of the micrometastatic colonies [13,14]. Then, the effects of the organ-specific “soil” on the early metastatic colonization competency [6] and the ability of the newly formed colonies to induce angiogenic switch [7] are the key aspects of cancer progression from the micro- to the macrometastatic stage [15,16]. As these factors eventually define the chances of cancer patients to survive, micrometastases are considered a promising treatment target [12]. 

In contrast to macrometastases, the early stages of the metastatic cascade, from homing to the angiogenic switch, are extremely difficult to detect and study under natural conditions, and even more so in the lab for several reasons [17,18]. Clinically, micrometastases are mostly asymptomatic [8]. At the same time, their size is mostly below the detection threshold of the current clinical non-invasive methods (~2 mm) [19]. Moreover, because of their small size and sparse distribution in the body, micrometastases can be easily missed, even by histology, particularly in the distant organs beyond the sentinel lymph nodes [8,17,18]. As a result, studies of such latent, early-stage post-extravasation cancer events are rare [6] and experimentally challenging [18,20]. This gap in micrometastases research cannot be bridged without reproducible and sustainable models of early metastatic colonization. For biological accuracy, these models should reflect several essential aspects of the micrometastases formation that are briefly discussed below.

The metastatic colonization of distant organs starts from and is particularly affected by the interactions between extravasated cancer cells and the resident extracellular matrix (ECM) [10,21,22]. A growing body of literature indicates the prominent role of the ECM’s composition and physical properties in controlling tumor progression (see [23] for a comprehensive review). The availability and size of cellular adhesion sites and stiffness [24], as well as confinement and porosity [25,26], significantly contribute to this regulation. These ECM properties vary greatly between different organs [22,27,28,29]. Then, the biologically relevant models of micrometastases should integrate cancer cells with the native organ-specific ECM [30,31,32]. Seemingly, such environments could be available via animal models. However, animal models of experimental or spontaneous metastases are rather suitable for the induction of macrometastases [13,33,34] that are amenable to contrast-enhanced medical imaging modalities [35]. Experimental metastases (induced by injections of cancer cells suspensions into the bloodstream) poorly reflect the human patterns of metastatic cancer organotropism [33]. Spontaneous (derived from the transplanted tumors) or genetically engineered animal models of metastases possess many limitations linked to the contribution of the immunocompromised microenvironment, the animal origin of the tumors, or the time course of the metastatic disease development. Overall, animal modeling is not ideal for the micrometastases simulation due to the low throughput capacity [36] (vs. a high cost). This is especially notable in drug development research, where large and reproducible numbers of representative colonies are needed. On the contrary, in vitro models such as conventional monolayers and 3D multicellular spheroids cannot reflect the micrometastatic biology and are missing the role of the ECM as the homing niche [37].

Tissue engineering provides a valuable tool for the reconstruction of organ-specific environments by using organ-derived ECMs as three-dimensional (3D) scaffolds for cell culture. Such scaffolds are obtained by decellularization (DCL). DCL involves the removal of cells from native organs, tissues, or cultured cell sheets [38,39,40,41,42]. Decellularized tissues are among the most clinically successful biomaterials in regenerative medicine, because they preserve three essential organ-specific features of the ECM: The composition, architecture, and biomechanical properties that support the desired tissue regrowth [43]. In vitro 3D tumor models on decellularized scaffolds hold great promise for cancer biology research, drug development, and nanomedicine [44,45,46].

The best quality of DCL (implying a low level of cellular residuals and a minimally affected ECM [47]) can be achieved by perfusion of whole organs via natural vasculature (whole-organ DCL, or WO-DCL) in a bioreactor [41,48] or in situ [49,50]. This requires a precise surgical technique with cannulation of the blood vessels in a live anesthetized animal, followed by ex vivo processing/preparation of the cannulated and extirpated organ. Unfortunately, WO-DCL is almost non-scalable (i.e., one animal, one organ, and one bioreactor place at a time) and therefore non-applicable in high-throughput applications. Alternatively, small fragments of organs and tissues can be decellularized by immersion in static or shaking baths, with the solutions able to destroy cellular membranes and remove cellular debris from tissues [42]. This well-established technology is widely used to prepare highly biocompatible collagen-based biomaterials for clinical applications [51]. However, further processing (e.g., freeze-drying and solubilization) of the decellularized materials obtained by immersion DCL is usually required to achieve reproducibility and scalability of biomaterial production [51,52,53,54]. Such processing destroys the tissue-specific architecture of the ECM, transforming it into a handy but artificial substrate. In addition, the sourcing of tissues for DCL remains an ethical, economic, and biosafety problem. In regenerative medicine, human cadaveric and discarded organs and tissues are the preferred but low-available materials for DCL [47]. As the ECM is evolutionary conservative across many animal species [55,56,57], there is an option to use organs and tissues, for example, from cows and pigs, to produce DCL scaffolds [52,58].

In the current study, we employed a DCL-based tissue engineering methodology to create a new organ-specific model of micrometastases applicable in vitro and in vivo. First, we reconciled the advantages of the WO-DCL and immersion DCL methods by introducing a new source of organs for DCL, such as chick embryos (CEs). The small size of the CE organs (up to 2–3 cm) allowed to achieve high-quality whole-organ DCL by immersion, while avoiding the need of laborious and time-consuming operations in conventional WO-DCL. We named our protocol iWO-DCL (“immersion–agitation whole-organ DCL”). It uses the same set of solutions for different organs of CEs, with parallel processing in shaking baths at room temperature under organ-specific time and shaking speed. The protocol does not require complex equipment and relies on the industry supplied poultry product (CEs) as a highly controllable, reproducible, and affordable source of organs for DCL. This allows ethical, labor-, and cost-efficient modeling of tumor micrometastases for cancer biology research and drug/nanomedicine development.

Herein, we applied iWO-DCL to create acellular organ-specific scaffolds (AOSSs) of CE brains, lungs, livers, hearts, small intestines, proventriculi and ventriculi, breast muscles, and spleens. Next, we seeded the obtained panel of AOSSs with the cells of one of the most metastatically aggressive human tumors, triple-negative breast cancer (TNBC). The obtained 3D tissue engineering constructs (TECs) were cultured in vitro for 1–4 weeks, simulating the formation of avascular micrometastases. Using TECs, we analyzed the role of organ-specific ECMs in the metastatic organotropism of TNBC and the mechanisms of the colonization of hepatic ECMs by breast cancer cells. Next, using a chick embryo chorioallantoic membrane (CAM) assay, we confirmed that the liver ECM-TNBC TECs could induce angiogenic switch in vivo. Finally, we evaluated the feasibility of our 3D tissue-engineered model of TNBC hepatic micrometastases as a testbed for drug and nanomedicine development.

## 2. Materials and Methods

### 2.1. Study Design

**Figure 1:**
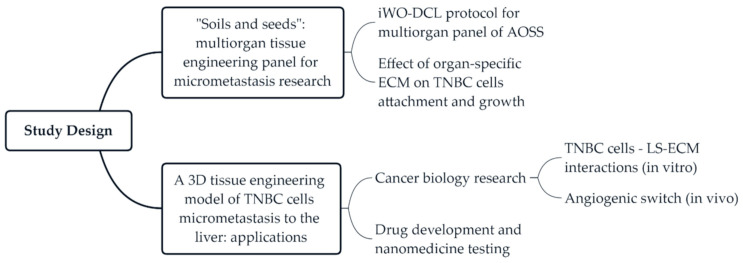
The design of the current study. Abbreviations: iWO-DCL—immersion whole-organ decellularization; AOSS—acellular organ-specific scaffolds; ECM—extracellular matrix; TNBC—triple-negative breast cancer; 3D, three-dimensional; LS-ECM—liver-specific ECM.

**Figure A1:**
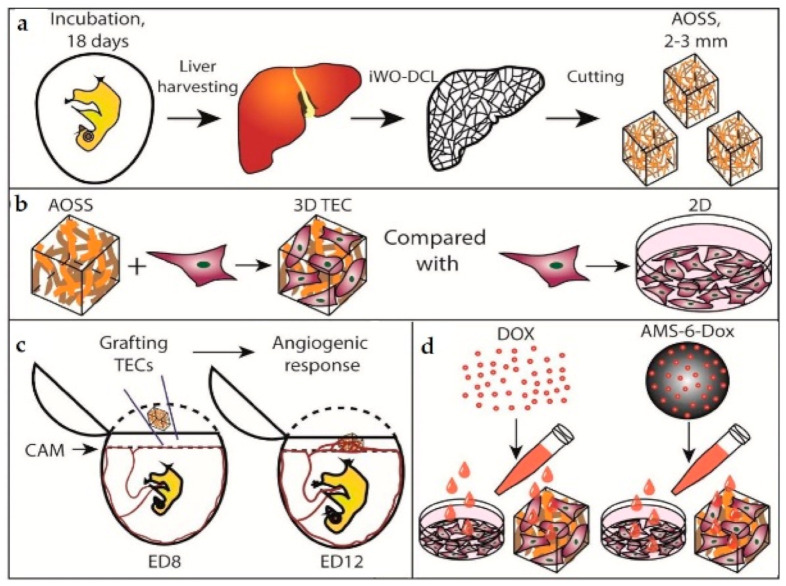
A scheme of the study design. (**a**) Obtaining the liver AOSSs: Incubation of CEs and harvesting livers from ED18 CEs, followed by iWO-DCL of the livers and preparation of the AOSSs by cutting decellularized livers into small fragments. (**b**) Preparation of 3D TECs by seeding of the liver AOSSs with MDA-MB-231 cells; analysis of the colonization patterns and cellular geometries in TECs and comparison of cell population dynamics in TECs with conventional 2D cell cultures by MTT assay and histopathological examination. (**c**) Evaluation of angiogenesis potential of the TECs using CAM assay. (**d**) Comparison of the effect of molecular Dox and nanoparticle-formulated AMS-6-Dox in a 2D culture of TNBC cells and in 3D TECs. Abbreviations: AOSS—acellular organ-specific scaffold; CE—chick embryo; ED—embryonic day (age of the chick embryo); iWO-DCL—immersion–agitation-assisted whole-organ DCL; DCL—decellularization; TEC—tissue engineering construct; MTT—3-(4,5-dimethylthiazol-2-yl)-2,5-diphenyltetrazolium-bromide; CAM—chorioallantoic membrane of CE; Dox—doxorubicin; AMS-6-Dox—mesoporous silica nanoparticles loaded with 20% doxorubicin; 2D and 3D—two-dimensional TNBC cell cultures and 3D TECs.

The current study includes two series of experiments (Figure 1). In the first series, we developed a protocol of DCL that is applicable to multiple CE whole organs, and tested the in vitro biocompatibility of the obtained AOSSs by seeding them with human TNBC cells. Next, to explore the “seeds and soil” hypothesis regarding the contribution of organ-specific ECMs to the preferential metastatic spreading of breast cancer cells to certain organs, we applied digital analysis of the histological images to examine and quantify the organ-specific patterns of cellular attachment and colonization of the AOSSs. The second stage of the study was focused on the interactions between TNBC cells and liver-specific ECMs presented as CE liver-derived AOSSs. This experimental series included evaluation of the feasibility of the proposed 3D tissue engineering model of TNBC micrometastasis to the liver for cancer biology research and for drug and nanomedicine testing. The detailed design of the second series of experiments is presented in Section Appendix A.1 and Figure A1 (Appendix A.1).

### 2.2. Chick Embryo Incubation and Organ Collection

This study was approved by the animal ethics committee protocols of Macquarie University (ARA # 2015/006) and the University of New South Wales (ACEC #19/103B). Fertilized chicken (*Gallus domesticus*, the broiler breed *Ross 308*) eggs were delivered from a local hatchery. After delivery, the eggs were rested for 3–4 h at room temperature and then incubated in a standard cradle-type laboratory poultry incubator (R Com MARU Max 190, Autoelex Co., LTD, Andong, South Korea) at 37.5 °C and 65%–70% humidity with hourly turn over until embryonic day 18 (ED18), which is 3 days before natural hatching. This period was sufficient for the histoanatomical development of the organ structure of a chick. On ED18, eggshells were opened by a cut on the blunt ends of eggs, and the embryo with embryonic membranes was extracted using forceps and immediately decapitated (this method of culling keeps the high quality of the tissues of the chicks [59]). Then, feathers were removed from the abdominal walls and thoraxes of the CEs, and the organs of interest were carefully extracted through a central section. The animal procedure used for the angiogenic assay on the CE chorioallantoic membrane (CAM) is described below in Appendix A (Section Appendix A.3.4).

### 2.3. Immersion Whole-Organ Decellularization (iWO-DCL)

The iWO-DCL was performed by our original immersion DCL method [36,60] with some modifications. The extracted CE organs, including the livers, lungs, hearts, ventriculi and proventriculi, brains, small intestines, spleens, breast muscles, and skins, were washed in sterile PBS and placed in 50 mL Falcone tubes, with 5–15 organs/per tube (depending on the organ’s size, for example, up to 5 livers per tube) filled with 35 mL of 0.1% solution of sodium dodecyl sulphate (SDS) in phosphate-buffered saline (PBS), then closed tightly and fixed horizontally on the platform of an orbital shaker. Then, the organs underwent shaking at a speed of 90–150 rotations per minute (rpm) with periodic aseptic changes of washing media for a fresh portion every 3 h during the first 12 h, and then every 6 h during the next 12 h. Afterward, the solution was changed daily until the organs became translucent, and the liquid media turned colorless and transparent. The total processing time ranged from 2 to 21 days depending on the organ (approximately 14–21 days for the livers, and 2–4 days for smaller organs and the embryo batch). Next, the processed organs were aseptically placed in sterile containers and washed with a 1% antibiotic–antimycotic solution (A-A) (#A5955, Sigma-Aldrich, North Ryde, NSW, Australia) in PBS (pH 7.0) under shaking (30–90 rpm) with periodical changing of A-A/PBS with fresh portions, until the washing media became transparent and colorless, with no observable tissue components or foam. The processing was performed at room temperature. An overview and the technical notes explaining further details of the iWO-DCL protocol are shown in Table A1 in Appendix A.2, and Supplementary Video S1 in Supplementary Materials.

The as-obtained scaffolds were stored in a fresh sterile 1% A-A/PBS solution in a fridge (+4 °C) until further use, which preserved their functionality as 3D culture substrates for at least one year. The iWO-DCL protocol presented here was tested for the Ross 308, Cobb 500, and several Rhode Island × White Leghorn crossbreed CEs (on at least a hundred eggs of each type). The best and most robust performance was achieved when using the broiler breeds (Cobb 500 and Ross 308), while individual stage- and organ-specific adaptations were required for the others, e.g., the layer chicken eggs.

### 2.4. Cell Culture

MDA-MB-231 (ECACC 92020424) cells were expanded by culture in complete culture medium (CCM) prepared from Dulbecco’s Modified Eagle’s Medium (#D8437, DMEM/F12/Ham medium, Sigma-Aldrich, North Ryde, NSW, Australia) supplemented with 10% fetal bovine serum (#12003C, FBS; Sigma-Aldrich, North Ryde, NSW, Australia) and 1% penicillin–streptomycin (PS; 10,000 U/mL; #15140122, Gibco) under standard conditions (37 °C, humidified, 5% CO_2_ gas atmosphere). The culture medium was changed every two days, and the cellular growth was controlled using a phase-contrast microscope and cell counting. According to the cell counting data, the average population doubling time of MDA-MB-231 cells (used in the experiments in passages 4 to 6) was approximately 34 h, with an average viability of ~98%. The same culture medium was used for all in vitro experiments, unless otherwise specified.

### 2.5. Recellularization of CE Acellular Organ-Sepcific Scaffolds (AOSSs) with MDA-MB- 231 Cells

Small fragments (approximately 2 × 3 mm) of decellularized CE organs/tissues were cut by a scalpel and put into 24-well flat-bottom tissue culture plates (#3524Costar, Corning, Cambridge, MA, USA). One milliliter of 0.1% peracetic acid solution (#77240, Sigma-Aldrich, North Ryde, NSW, Australia) in 4% ethanol was added to every well for 2 h. Then, this solution was removed, and the AOSSs were washed with sterile PBS (0.4 mL per well) twice and sterilized by ultraviolet light in a tissue culture hood for 45 min (with turning the scaffolds with sterile tweezer twice to expose different sides). Next, each well was refilled with 1 mL of CCM. Following this, the plates with the AOSSs were placed into a tissue culture incubator and conditioned overnight under a humidified atmosphere with 5% CO_2_ at 37 °C. This step served for the scaffolds’ conditioning and an additional check of sterility.

MDA-MB-231 cells (1 × 10^5^ cells in a 20 µL drop of CCM) were seeded on the top of the AOSSs obtained by iWO-DCL of the CE livers, lungs, hearts, ventriculi and proventriculi, brains, small intestines, and breast muscles to form 3D TECs. One TEC was placed into each well of a 24-well culture plate. Control scaffolds were left unseeded. Next, the cells were allowed to attach to the substrates for 2 h in a tissue culture incubator, then added to with 1 mL of CCM per well and cultured for 1–28 days. On day 1, the TECs were carefully relocated to the new multiwell plates to preserve only the cell populations attached to the scaffolds. The CCM in the growing 3D cultures was carefully changed twice a week. For microscopy, viability assays, and histological analysis, the TECs were sampled on week 1 (the multiorgan panel of TECs based on CE livers, lungs, hearts, ventriculi and proventriculi, brains, small intestines, and breast muscles) or weeks 1, 2, 3, and 4 after seeding (liver-based TECs).

### 2.6. Analysis of the Structural Evolution of TECs

#### 2.6.1. Histology, Histomorphometry, and Fluorescence Microscopy

The AOSSs and TECs were fixed in 10% neutral buffered formalin, dehydrated in a graded series of alcohols, embedded in paraffin wax, and cut into serial sections of 5 µm in thickness by a rotary microtome. After deparaffination, slices were stained with hematoxylin and eosin (H&E), Van Gieson’s picrofuchsin, Masson’s trichrome, and toluidine blue, following conventional protocols. Stained histological preparations were examined using an upright research microscope Axio Imager Z2 (Zeiss, Oberkochen, Germany) equipped with dry-air EC Plan-Neofluar (5×/NA0.16; 10×/NA0.30; 20×/NA0.50 Ph) and an oil-immersion Plan Apochromat (100×/NA1.46 oil) objectives (Zeiss, Oberkochen, Germany). The relative cellularity of the TECs was examined using ImageJ software via color intensity-based segmentation of the area occupied by cells relative to the total section area of the TEC on the images of H&E-stained samples. The images were recorded using a preinstalled microscope digital video camera AxioCam (1388 × 1040, Zeiss, Oberkochen, Germany) in single-frame and stitching modes using Zen 2012 software. For the rapid check of the quality of DCL and the viability of the cells in the TECs, epifluorescence microscopy was performed. For the DCL quality check, the deparaffinized sections of AOSSs were stained with DAPI (#D9542, Sigma-Aldrich, North Ryde, NSW, Australia) to detect nucleic acids and with Phalloidin-TRITC (#P1951, Sigma-Aldrich, North Ryde, NSW, Australia) to detect f-actin, following the manufacturer’s protocol. For the live–dead cell detection, the TECs were stained immediately with fluorescein diacetate (#F7378, FDA; Sigma-Aldrich, North Ryde, NSW, Australia) and propidium iodide (#P4864, PI; Sigma-Aldrich, North Ryde, NSW, Australia) according to Application Note #33 by Ibidi GmbH (Germany) [61], counterstained with DAPI and imaged with the use of the filter settings for DAPI, FITC (for FDA), and PI on the same microscope.

#### 2.6.2. Scanning Electron Microscopy

The samples of AOSSs and TECs were fixed in 2.5% buffered glutaric aldehyde, further dehydrated in 70%–100% alcohols and contrasted by OsO_4_ using the Pelco Biowave Pro+ microwave processing system (PELCO BioWave Pro, Ted Pella Inc., Redding, CA, USA), before undergoing critical point drying in Tousimis Autosamdri-815 Critical Point Dryer (Tousimis research corporation, Rockville, MD, USA). Afterward, the samples were mounted on stabs with conductive carbon/graphite paint (ProSciTech, Kirwan, QLD, Australia) and coated with platinum using an Emitech K575x Pt sputter coater (Emitech Ltd., Ashford, Kent, U.K.). Electron microscope images were taken using Nova^TM^ NanoSEM 230 (FEI company, Hillsboro, OR, USA), which is a field emission scanning electron microscope, under an accelerating voltage of 5 kV, a work distance of 20 mm, and a size point of 30 in the secondary electron imaging mode.

#### 2.6.3. Atomic Force Microscopy

AFM imaging of the deparaffinized/non-coverslipped dry histological tissue (CE liver AOSSs) sections on glass slides was performed in air in the semi-contact mode (according to the method established by us earlier [62] with the modifications indicated below). The scans were taken following the selection of the regions of interest under bright field microscopy. The following regions of interest were explored in the CE liver AOSSs: (1) In the parenchymal compartment—the parenchymal ECM of former Disse’s space, the former parenchymal ECM in the vicinity of the vein and arterial walls; and (2) in the stromal compartment—the former walls of the arteries, veins, and venules.

The AFM measurements were performed on the Bruker Dimension ICON SPM. The DMT modulus was measured using the peak force tapping mode with the OTESPA-R3 probe (Bruker AFM probes). For accurate modulus measurements, the probe was calibrated according to the following procedure. First, the probe was withdrawn from the sample surface by at least 5 mms, so the spring constant could be calculated using the thermal tunning method. Then, the probe was engaged on a hard surface, namely, sapphire, with a engage set point of 0.5 V, and then the probe was ramped on the surface with 0.2 V of deflection to calculate the deflection sensitivity, which was determined to be 67.3 nm/V (an average of 5 measurements are taken per location and two locations were measured). After the ramp curves were done, the probe was calibrated again, and then the spring constant was updated to a more accurate value—in this case, 28 N/m.

The tip radius of the probe was checked with a titanium roughness check; this is a sample with sharp titanium flakes to check the tip radius. A slow scan rate of 0.2 Hz was used, as the features of the titanium sample were sharp and rough, which could damage the probe apex if the scan rate was too fast. After all the constants were determined, the probe was engaged on the tissue sample, and the scanning parameters, such as scan rate, peakforce setpoint, and feedback gain, were optimized accordingly, depending on the scan size and the scanning area. The resolution of the image was kept at 256 samples/line. The following is an example of the typical scan parameters that were used: Scan size, 5 × 5 µm; scan rate, 0.25–0.4 Hz; feedback gain, ~14; peakforce setpoint, 70 nN; Poisson ratio, 0.45. The data visualization and analysis of stiffness and surface roughness were performed using free Gwyddion 2.55 software (Czech Metrology Institute, Jihlava, Czech Republic). The statistical calculations were conducted using SPSS 26.0, as described below.

#### 2.6.4. Image Analysis for Histological Morphometry

Image processing techniques were applied to evaluate the cellular distribution and shapes in H&E-stained histological images of the evolving CE liver and human TNBC TECs acquired on weeks 1, 2, 3, and 4 of the in vitro culture. During pre-processing, Gaussian blur with s.d. σ = 0.65 μm was applied to reduce high-frequency components. Using color deconvolution, the images were split into three separate channels containing cells, a matrix, and a background, respectively. Next, images with cells were thresholded and a segmentation algorithm was performed, where it was possible to extract single cell boundaries. The cell shape was described by calculating the circularity of a convex hull. The reason for using convex hull approximation of cell boundaries is that it reduces the variability of the data and allows more accurate classification. The boundaries of the parenchymal and stromal compartments were outlined manually. Image processing was performed using ImageJ and MATLAB 2016b.

#### 2.6.5. MTT Assay for Evaluation of the Population Evolution of MDA-MB-231 Cells in 2D and 3D TECs

The cell viability was tested in 3D liver TNBC TECs and matching 2D cell cultures of MDA-MB-231 cells using a modified MTT colorimetric assay. This assay relies on the reduction and conversion of yellow 3-(4,5-dimethylthiazol-2-yl)-2,5-diphenyltetrazolium-bromide (MTT) reagent (#M2128, Sigma-Aldrich, North Ryde, NSW, Australia) into purple formazan salt, where the optical absorbance of formazan crystals dissolved in dimethyl sulfoxide (DMSO) represents the activity measure of mitochondrial dehydrogenase [63]. The tested cultures were grown in CCM in a humidified atmosphere under 5% CO_2_ at 37 °C.

The following procedure was introduced to ensure an appropriate comparison between 2D and 3D TEC cultures. The fifth passage MDA-MB-231 cells were seeded on chick embryo liver AOSSs, as described earlier, while the same amount of the cells (1 × 10^5^ in a 30 µL drop of CCM) was deposited in the middle of the wells of a 24-well culture plate (Costar, Corning, Cambridge, MA, USA) to perform high-density seeding. Next, the cells in both cultures were allowed to attach to the substrates for 2 h in a tissue culture incubator in a humidified atmosphere under 5% CO_2_ at 37 °C, and then filled with 1 mL of CCM per well and cultured for 1 day.

After 24 h, the media were removed, and the samples were washed twice with PBS to eliminate unattached cells. Next, the 3D TECs were aseptically transferred to new 24-well culture plates to get rid of the cells adhered to the plastic and not to the scaffolds in the original cultures, then filled with fresh CCM (1 mL per well) and cultured for 4 weeks, as described above. At the same time, 2D cell cultures, after washing with PBS, were filled with CCM, and cultured for 4 weeks, without splitting. The media were changed twice a week in both types of cultures.

The MTT assays were carried out on days 1, 7, 14, 21, and 28 after seeding (day 1 and weeks 1, 2, 3, and 4, respectively). For each assay, 3 samples of TECs were randomly selected and transferred to a separate 24-well plate for testing, while 3 wells of cells growing in a 2D culture were used as the internal control. After double washing with PBS, 500 µL of MTT reagent (0.5 mg/mL in the phenol red free cell culture medium; DMEM/F12; #D6434, Sigma-Aldrich, North Ryde, NSW, Australia) was added to each well. Then, the samples were incubated at 37 °C in a tissue culture incubator for 4 h to allow precipitation of insoluble formazan crystals. After this, the supernatant was carefully collected, and 500 μL of DMSO was added to the wells and left for 15 min in the dark on a rocking platform at room temperature to dissolve purple formazan crystals. Next, four portions of 100 µL of the dissolved MTT product was taken from each well, transferred to separate wells of a clear 96-well culture plate (#3585, Costar, Corning, Cambridge, MA, USA) and used for absorbance measurements. The samples’ absorbance was measured in a spectral band centered at 570 nm by a PHERAstar multiplate reader (BMG Labtech, Ortenberg, Germany), with unseeded wells used as blank controls. The experiments were repeated twice, with at least triplicates for each condition; the results were corrected for the blank controls by MARS Data Analysis software (BMG Labtech, Ortenberg, Germany).

#### 2.6.6. Modeling Cell Growth Dynamics

The logistic growth model was fitted to the cell viability values at each point in time in the 2D and 3D cultures (Equation (1)):

(1) $$N=\frac{C_{0}C_{max}}{C_{0}+\left(C_{max}-C_{0}\right)e^{-dt}},$$

where *N* is proportional to the number of viable cells at time t; *C*_0_ is proportional to the number of viable cells at the start; *C_max_* is proportional to the maximum number of viable cells; and *d* is the growth rate. The fit was performed using the nonlinear least squares function in MATLAB software.

### 2.7. Evaluation of Angiogenic Potential of Liver TNBC TECs in Vivo (CAM Assay)

#### 2.7.1. Grafting and Imaging Procedures

The detailed description of the used procedures of the angiogenic assay on CE CAM [64] can be found in Appendix A.3.4, Appendix A.3.4.1, Appendix A.3.4.2 and Figure A11 and Figure A12. Briefly, liver TNBC TECs, CE liver AOSSs, or MDA-MB-231 cell suspensions were grafted on CAMs separately, one sample of each material type per egg, 5 replicates per a group. Before grafting, the TECs were cultured in vitro for 12 days, as described above, reaching the stage when they contained approximately 2 × 10^5^ viable cells (according to MTT assay data). The liver AOSSs were kept in CCM for 24 h before grafting on CAM. The suspension of 2 × 10^5^ MDA-MB-231 cells in 60 µL of CCM were grafted on CAMs within a sterile rubber ring. The angiogenic effect induced by the TECs, liver AOSSs, and suspensions of MDA-MB-231 cells in PBS grafted on CAMs was evaluated by stereomicroscopy imaging performed on the day of grafting (embryonic day 8, ED8) and on ED12, in comparison to the natural growth of blood vessels of CAM occurring during the same period of chick embryo development.

#### 2.7.2. Angiogenesis Quantification

The images of CAMs taken using an 2×/0.5 N.A. objective were processed using the following methodologies to evaluate the dynamics of the vascular length density and branching of blood vessels in CAMs. For the control and cell groups, 10 regions of interest (ROIs) per egg were chosen manually. CAMs grafted with 3D-engineered tumors (TECs) and liver AOSSs (scaffolds) were imaged from the 4 corners around the graft, and then 3–4 ROIs were chosen from every corner to obtain approximately 10 ROIs per egg. Since the vessels have higher contrast in the green channel, the RGB images were split to obtain the green component. Histogram stretching of the grey-level intensities was performed to enhance the image contrast. Then, a ridge detection algorithm [65] involving convolution with the derivatives of a Gaussian smoothing kernel was used to capture the blood vessels by finding the local minima, resulting in a skeleton of a vascular pattern. For each ROI, the total branch length of the obtained skeleton was divided by the area of the ROI, and then the results were averaged to obtain the mean vascular density per egg. The exploration of the branching parameters of the vascular trees was performed using the Angiogenesis Analyzer plugin for ImageJ [66] (see Appendix A.3.4, Appendix A.3.4.3 for the details). Image processing was performed using ImageJ and MATLAB 2016b.

### 2.8. Cytotoxicity and Cellular Uptake of Doxorubicin (Dox) and Mesoporous Silica Nanoparticles Loaded with Dox in 3D Liver TNBC TECs and in Matching 2D Cultures of TNBC Cells

#### 2.8.1. Preparation of Mesoporous Silica Nanoparticles and Their Loading with Dox

Anionic surfactant-templated mesoporous silica nanoparticles (AMS-6), reported by us earlier [67,68], were synthesized in-house following a protocol described elsewhere [68]. Briefly, *N*-lauroyl-l-alanine was used as surfactant, and APES was applied as a costructure directing agent to achieve connected pores in TEOS-sourced silica nanomaterial. The sample was calcinated at ~550 °C using a temperature gradient of 1.5 °C/min to remove surfactant. Next, as-synthesized mesoporous silica nanoparticles (AMS-6) were loaded with 20% Dox (#D1515, doxorubicin hydrochloride, Sigma-Aldrich, North Ryde, NSW, Australia). Dox diluted in 100% ethanol was added to AMS-6 nanoparticles in a round-bottomed flask mounted on a rotary evaporator, and ethanol was evaporated at 40 °C under vacuum with slow rotation. The collected sample was air-dried overnight.

#### 2.8.2. Characterization of Mesoporous Silica Nanoparticles

Unloaded (“pure”) AMS-6 and Dox-loaded AMS-6 (AMS-6-Dox) were characterized using transmission electron microscopy (TEM), X-ray diffraction (XRD), thermogravimetric analysis (TGA), and dynamic light scattering (DLS). Nitrogen adsorption/desorption isotherm measurements were carried out to evaluate the effective surface area of the AMS-6 and AMS6-Dox samples. For TEM sample preparation, a small amount of dry AMS-6 or AMS-6-Dox was thoroughly crushed in a mortar and then diluted in ethanol. A drop of the suspension was placed on a copper grid and dried. Next, the grid was placed in a sample holder of a JEOL 3000F TEM (Peabody, St. Louis, MO, USA) and imaged at 300 kV with the resolution of 1.6 Å. Images were obtained using a Gatan SC1000 11-megapixel CCD camera (Gatan Inc., Pleasanton, CA, USA), with a 1024 × 1024 pixel Gatan image filter (Gatan Inc., Pleasanton, CA, USA). XRD measurements of 20 mg of the dried nanoparticle samples were carried out using XRD instrument Bruker D8 Discover equipped with VÅNTEC-500 detector featuring a 140 mm diameter window (Bruker Corporation, Billerica, MA, USA).

XRD patterns were recorded using the Cu Kα anode (λ = 0.1542 nm), operating at 40 kV and 30 mA. TGA measurements were performed using a 1 mg sample placed in an aluminum crucible TGA2050 (TA Instruments, New Castle, DE, USA) and heated from 25 to 850 °C at 10 °C/min under air flow at 10 mL/min. Nitrogen adsorption/desorption isotherms were acquired at a temperature of –196 °C using liquid nitrogen with a TriStar II by the Micromeritics^®^ instrument (Micromeritics Instrument Corporation, Norcross, GA, USA), following degassing of the AMS-6 and AMS-6-Dox samples under vacuum using a VacPrep™ 061 by Micromeritics^®^ instrument (Micromeritics Instrument Corporation, Norcross, GA, USA) for ~10 h at 120 °C. The surface area was calculated using the Brunauer–Emmett–Teller (BET) equation [69].

Hydrodynamic diameters and the zeta-potentials of colloidal AMS-6 and AMS-6-Dox were measured in PBS and CCM (1 mg/mL) by Zetasizer Nano ZS (Malvern Panalytical Ltd, Malvern, UK) in three runs followed by averaging.

#### 2.8.3. MTT Viability Assay of 2D and 3D TEC Cell Cultures Treated with Free and Nanoformulated Dox

The TECs were cultured for 3 weeks as described above. For the control 2D in vitro culture, MDA-MB-231 cells were seeded in 96-well plates (#3599, Costar, Corning, Cambridge, MA, USA) at a density of 2 × 10^4^ cells per well and incubated in CCM for 24 h before the test. Then, the culture medium was removed from all the cultures, the TECs were aseptically transferred to new 24-well culture plates, and all of the cultures were washed 3 times with PBS. Free Dox of the concentrations ranging from 0.1 to 10 µg/mL and AMS-6 (50 µg/mL) and AMS-6-Dox of the concentrations ranging from 0.5 to 50 µg/mL (Dox-equivalent, 0.1–10 µg/mL) were diluted in CCM and sonicated immediately before the test. Each concentration of each type of the tested compounds was applied in a total volume of 100 µL to 8 wells of 96-well plates for challenging of the 2D culture of MDA-MB-231 cells (2 biological replicates × 4 technical replicates). At the same time, 6 TECs growing in 24-well plates were used to test each concentration of each compound, and the added volume of the dispersions was 400 µL per well (2 biological replicates × 3 technical replicates). The 2D and 3D cultures treated with CCM were used as a control. The exposure time was 36 h. Then, MTT tests were performed, as described above (see Section 2.6.5). The supernatant was removed and 100 or 400 µL of DMSO was added to the wells of 96-well plates (2D cultures) and 24-well plates (3D TECs), respectively. The solution of formazan in DMSO from the TECs was transferred to the new 96-well plate (100 µL per well; 3 samples per TEC), and the absorbance of the tested cultures was measured. The mean percentage of dead cells *E* and the standard deviation *σ_E_* were recalculated based on the absorbance of the controls using Equation (2):

(2) $$E=\left(1-\frac{A}{A_{0}}\right)\times 100\%,\;\sigma_{E}=\left|\frac{100}{A_{0}}\right|\sigma_{A},$$

where *A* is the average absorbance in each group, *A*_0_ is the average absorbance of the corresponding control, and *σ_A_* is the standard deviation of the absorbance in each group.

#### 2.8.4. Pharmacodynamics Modeling

The experimental measurements of the percentage of dead cells in the 2D and 3D cultures after administration of free or nanoformulated Dox were described by the following sigmoid Equation (3):

(3) $$E=\frac{E_{max}}{1+10^{\frac{EC50-dose}{h}}},$$

where *E_max_* is the maximum effect (%), *EC*50 is the half maximal effective concentration (µg/mL), *dose* is the concentration of administered Dox (µg/mL), and *h* is the Hill coefficient. The half maximal inhibitory concentration (IC_50_), i.e., the drug concentration needed to obtain 50% of cell death, was calculated in each group using the line of the best fit. The parameters of the model are provided in Table A6 and Table A7, respectively, for free and nanoformulated Dox (Appendix A.3.5.2).

The fit was performed using a weighted unconstrained nonlinear curve fit (MATLAB r2016b) with the inverse error as the weight, and the dose values were first logarithmically transformed. The 0% cell death (100% viability) at 0 µg/mL in controls was given a higher weight as the data were normalized to these values and the model is expected to approach this intercept closely.

#### 2.8.5. Confocal Microscopy Study of the Uptake of Free and Nanoformulated Dox in 3D TECs and 2D Cultures

As-produced liver TNBC TECs were cultured for 3 weeks. Control 2D in vitro cultures of MDA-MB-231 cells were seeded onto sterile coverslips placed into wells of a 24-well plate (Costar, Corning, Cambridge, MA, USA) at a density of 5 × 10^4^ cells/well and incubated under standard conditions in 1 mL of CCM during 24 h prior to the observation. Dox solution (10 µg/mL), AMS-6 nanoparticle (50 µg/mL), and AMS-6-Dox nanoparticle (50 µg/mL; Dox-equivalent, 10 µg/mL) dispersions were prepared as described above. Next, after removal of the culture media and triple washing with PBS, the tested compounds were added to the wells with 2D and 3D cultures in a total single volume of 0.5 mL per well. The wells added to with CCM without Dox or nanoparticles were used as controls. The prepared 2D and 3D cultures were incubated for 24 h in a tissue culture incubator at 37 °C and 5% CO_2_; next, following thorough rinsing 3 times with PBS to remove free Dox and nanoparticles, they were fixed with 10% neutral buffered formalin at room temperature.

After a 24 h fixation process and another washing with PBS, the samples were stained with DAPI solution in PBS (#D9542, Sigma-Aldrich, North Ryde, NSW, Australia) for 20 min at 37 °C. Microtome sections (6 µm in thickness) of the frozen 3D TECs were prepared and also stained with DAPI. Next, the staining solution was removed, and the coverslips were washed twice with PBS to eliminate unbound DAPI. Finally, the samples were mounted on glass slides with Dako anti-fade mounting media and sealed with nail polish.

Dox and AMS-6-Dox cellular uptake was imaged by an inverted Zeiss LSM 880 laser scanning confocal microscope (Zeiss, Oberkochen, Germany), using a Plan Apochromat 10×/0.45 N.A. M27 and Plan Apochromat 40×/1.3 N.A. oil DIC UV-IR M27 objectives. Dox fluorescence was observed using 488 nm excitation and 535–673 nm emissions, and DAPI fluorescence was observed using 405 nm excitation and 411–528 nm emissions.

### 2.9. Statistical Analysis

The data are expressed as means ± standard deviations (SD), and the 95% confidence intervals (CI_95%_) for the means were calculated. Due to the non-Gaussian nature of the data, the two-sided Mann–Whitney *U*-test was used to evaluate the significance of intergroup differences between the means. The two-sampled Kolmogorov–Smirnov test was used to compare any two observed distributions of branch length density. Statistical significance was reported as follows: * *p* < 0.05, ** *p* < 0.01, and *** *p* < 0.001 or the exact *p*-value was provided where possible. Statistical analyses were performed using R Statistical and SPSS 26.0 software.

## 3. Results

### 3.1. Soils and Seeds: Development of a Chick Embryo-Based Multiorgan Tissue Engineering Platform for Modeling Cancer Micrometastases

**Figure A2:**
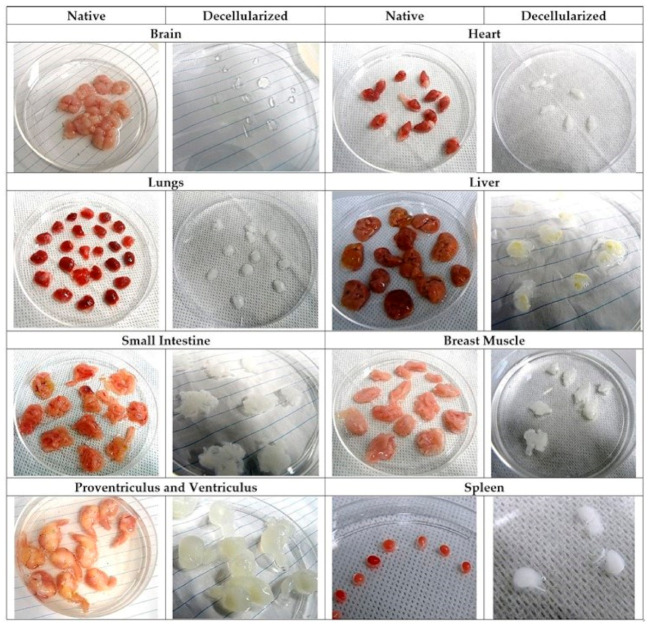
Effect of iWO-DCL on the macroscopic appearance of ED18 CE organs at the beginning and the end of processing. Note loss of natural color, whitening of the tissue, and increase in transparency. Some organs, such as the brain, heart, lungs, liver, and skeletal muscles, also significantly decreased in their volume. The samples of the small intestine and spleen shown in the “Native” columns represent the organs that were processed for DCL for 4 h to exemplify the gradual loss of the red tone starting from the edges of the organs due to the combined effects of diffusion and convection of the detergent that destroys the organ cells. This centripetal pattern of discoloration is commonly observed in the organs undergoing iWO-DCL.

iWO-DCL-induced significant macroscopic changes of organs emerged as a gradual loss of native color toward whiter and more translucent appearance and the volume reduction (Figure A2 in Appendix A.2). The time of decellularization varied depending on the organs’ original volume and density (see Table A1 in Appendix A.2). In originally non-translucent organs, it was notable that during iWO-DCL, the discoloration developed centripetally: It started from the edges of the tissue and then spread to the rest of the organ in a patchy way, and later converted into a diffuse whitish color and semitransparency. The majority of decellularized organs, except the brain, preserved the shape reflecting to the original one, while the matrix loosened to a certain extent.

**Figure 2:**
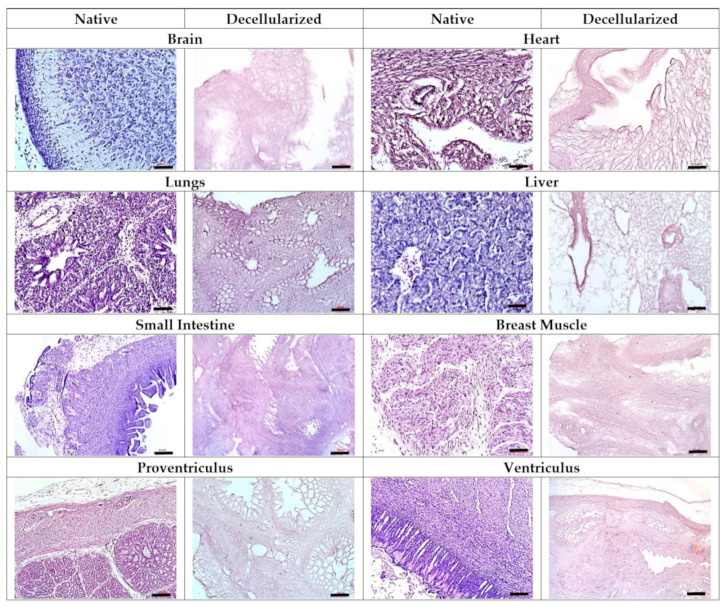
Effect of iWO-DCL on various organs of ED18 CEs. Note complete removal of the cells. The specialized compartments of the organs are recognizable in the matching AOSSs, except the brain AOSSs, where only the ECM areas of different density, with more fibrous (on the left) or more sponge-like (on the right) structures can be seen. In the heart the AOSSs, the myocardium and endocardium compartments are discernible. In the lung AOSSs, the specialized avian pulmonary architecture is preserved, with clearly visible ECMs of parabronchi, alveolar, air capillaries, and the interparabronchial septae. In the liver AOSSs, the elements of former parenchyma (mostly Disse’s space ECMs) and stroma (central and elements of triads) are present. In the small intestine AOSSs, the histoanatomically preserved ECMs of all conventional tissue layers of the organ are visible. The breast muscle AOSSs contain the interchanging areas of the denser and aligned and looser ECM elements corresponding to the distribution of the muscular bands, as well as the ECMs of arteries and veins. The AOSSs obtained from the proventriculi and ventriculi demonstrate excellent preservation of the histoanatomy of the organ layers and glandular elements. Staining with Hematoxylin and Eosin. Scale bars, 50 µm (brains, hearts, lungs, livers, and breast muscles) and 100 µm (small intestines, proventriculi, and ventriculi).

Histological examination confirmed the complete removal of the cellular material by iWO-DCL in all studied types of CE organs. The ECM generally preserved its organ-specific architecture (Figure 2).

**Figure 3:**
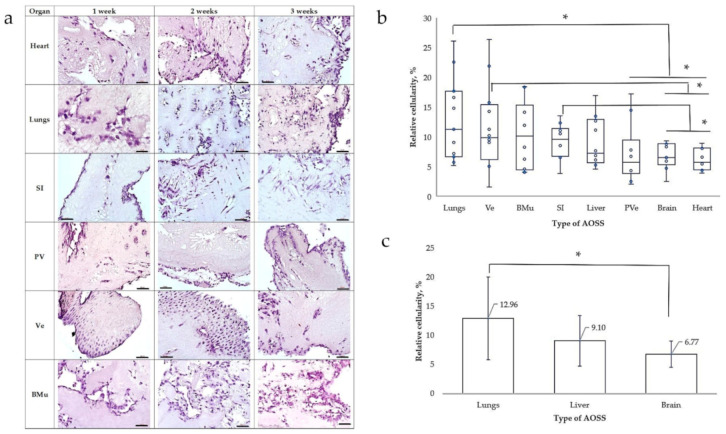
(**a**) Structural evolution of the organ-specific TECs that combine human MDA-MB-231 cells and CE AOSSs, as observed histologically during 3 weeks of in vitro culture. All of the TECs demonstrate initial colonization of the surface areas with cancer cells, followed by progressive invasion into the deeper parts of the scaffolds with increasing total cellularity. Note the organ-specific colonization patterns such as cells’ growth in association with the luminal ECM elements, such as the former walls of blood vessels in the heart and BMu, and colonization of parabronchial ECM in the lung TECs; linear pattern (“Indian-files”) of tumor invasion along the former muscular layers in SI ECM and the tunica muscularis in Ve; and the templated colonization of the former glandular elements and secretory lining of PV and Ve. Staining with Hematoxylin and Eosin. Scale bars, 50 µm (brains, hearts, lungs, SI, and BMu) and 100 µm (PV and Ve). (**b**) Relative cellularity of the TECs combining MDA-MB-231 cells and AOSS obtained from the different organs of origin on the 7th day of in vitro culture. Blue circles indicate the individual values, the blue horizontal lines show the mean, the boxes represent the range between the 25th and 75th percentiles, and the whiskers reflect the minimal and maximal observed values. Statistically significant differences (* *p* < 0.05) between the studied TECs are shown by black lines. (**c**) Relative cellularity (mean ± standard deviation) of the MDA-MB-231/AOSS TECs representing the common cites of the distant organ metastases of breast cancer. Statistically significant difference (* *p* < 0.05) between the lung and brain TECs is shown by a black line. Abbreviations: SI—small intestine; PV—proventriculus; Ve—ventriculus; BMu—breast muscle.

All obtained types of CE AOSS were successfully recellularized with human MDA-MB-231 cells and formed viable 3D TECs, indicating the biocompatibility of the AOSSs, efficient cellular adhesion, and notable proliferation on the matrices, observed for at least one week. Furthermore, in the longitudinal experiments, the TECs combining several types of CE AOSSs and TNBC cells were confirmed to be able to successfully survive for at least two to three weeks in vitro (data not shown), indicating the potential for the detailed studies on the interactions of the organ-specific ECMs and cancer cells. Importantly, MDA-MB-231 cells demonstrated organ-specific patterns of colonization of the ECMs originating from different organs (Figure 3a).

Figure 3b,c illustrate the results of the “soils and seeds” test, showing the effect of organ-specific ECMs on the relative amount of cells able to attach and grow on these substrates. The highest relative cellularity was observed in TECs formed by CE lung AOSSs and MDA-MB-231 cells. This was followed by the TECs containing TNBC cells and the CE AOSSs derived from the ventriculi, breast muscles, small intestines, livers, proventriculi, brains, and hearts. At the level of statistical significance, the lung ECMs supported higher cellularity than the ECMs of the proventriculi, brains, and heart matrices; the ECMs derived from ventriculi were more cellularized than the ECMs of the brains and hearts, while the small intestine ECMs maintained a higher cell density than the AOSSs prepared from the CE brains and hearts. Moreover, statistically, equally efficient colonization by TNBC cells was observed in the AOSSs obtained by iWO-DCL of the CE lungs, ventriculi, breast muscles, small intestines, livers, and proventriculi, indicating statistically equal risk of the initiation of the TNBC micrometastases that could be attributed to the role of the organ-specific ECMs, with a few exceptions. In particular, our results show that the ECMs of lungs may contribute more strongly to the increased risk of the secondary tumors than the brain ECMs, while the liver ECMs had an intermediate position between these two organs (see Figure 3c).

### 3.2. Three-Dimensional Tissue-Engineered Model of TNBC Micrometastatsis to the Liver for Cancer Biology Research

In the next stage of the study, we concentrated on the interactions between the liver-specific ECM (LS-ECM) and TNBC cells in 3D TECs and on applications of this tissue engineering model in cancer research.

#### 3.2.1. IWO-DCL Reveals Compartmental Organization of LS-ECM

**Figure 4:**
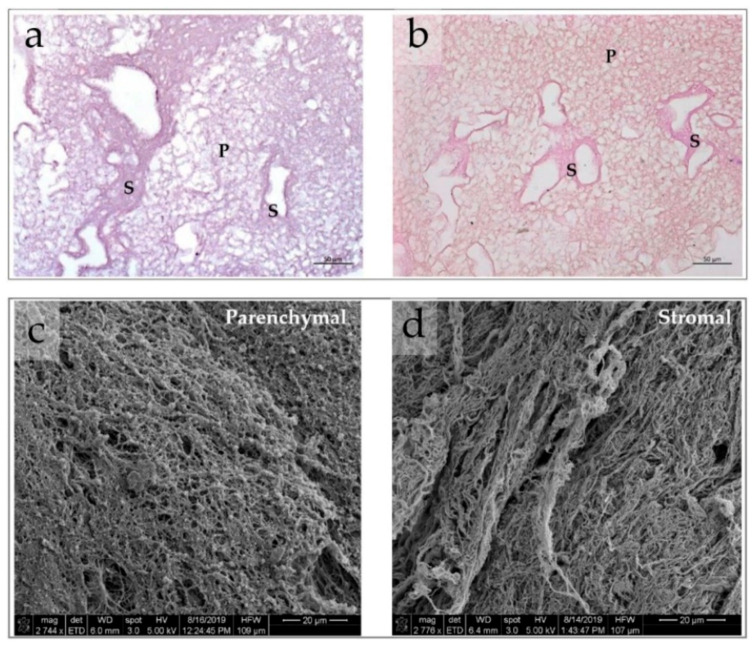
(**a**,**b**) Histological features of CE liver AOSSs. Complete removal of the cells and compartmental structure of the liver ECMs is clearly visible in samples stained by the hematoxylin and eosin (**a**) and Van Gieson’s (VG) (**b**) methods. The elements of the parenchymal (labeled “P”) and stromal (labeled “S”) compartments of the CE liver ECMs. The ECM of the parenchymal compartment is loose and mesh-like, with randomly oriented matrix elements of the former Disse’s space; while the ECM of the stromal compartment is denser, with more aligned and more fuchsinofilic (pink-red when stained by the VG method). The VG staining reveals the collagenous nature of the liver AOSSs and indicates the higher contents of fibrous/crosslinked collagen in the stromal compartment in comparison to the parenchymal one. Scale bars, 50 µm. (**c**,**d**) SEM images of CE liver AOSSs. Scale bars, 20 µm. The fragments representing parenchymal (**c**) and stromal (**d**) compartments are shown. Parenchymal compartment is characterized by a fine, random, mesh-like structure, while the stromal LS-ECM is composed of coarser and more parallel aligned fibrillar elements.

**Figure A3:**
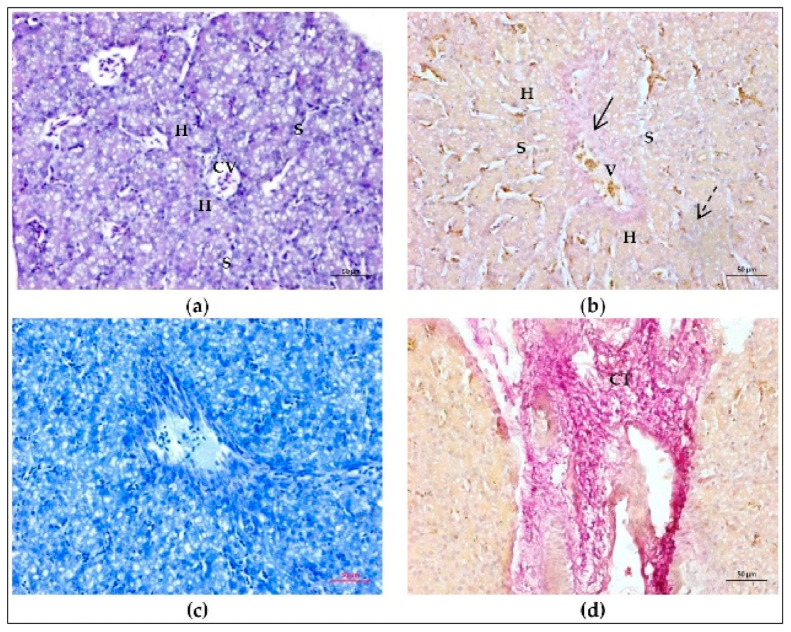
Histological structure of intact chick embryo liver extracted on ED18. (**a**) Hematoxylin and eosin staining reveals cords of hepatocytes (H) separated by narrow voids of sinusoids (S) arranged around the central vein (CV). The red blood cells in chick embryos are ellipsoid in shape and contain nuclei stained dark by hematoxylin. These cells are visible in the sinusoids and vein lumens. (**b**) In the samples stained by the Van Gieson’s method, the radial structure of hepatic lobule is more clearly discernible. Note pink staining of collagen (arrow) in the wall of a large vein (V) and more diffuse pink staining in the parenchymal part of the tissue (dashed arrow). (**c**) Orthochromatic staining of the liver tissue by toluidine blue indicates a low content of acid glycosaminoglycans. (**d**) Subcapsular area of the liver. Note connective tissue (CT) of the liver capsule and interlobular septae discernible by bright fuchsinofilia in samples stained by the Van Gieson’s method. Strong fuchsinofilic staining reflects high concentration of mature fibrillar collagen elements. Scale bars, 50 µm.

**Figure A4:**
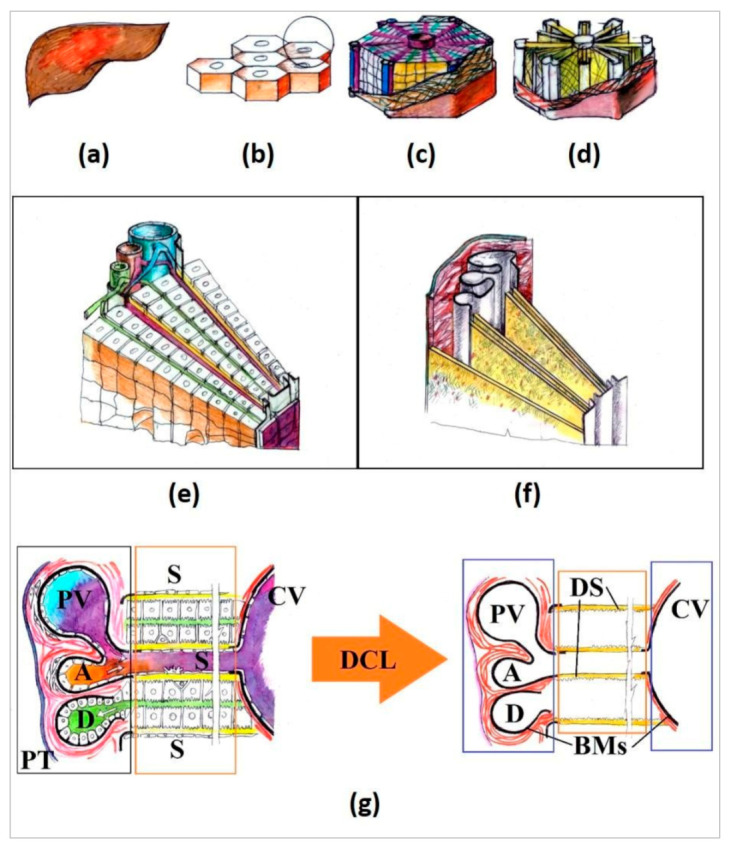
Schematics of the liver structure and the effect of DCL on its constituents. The liver (**a**) is formed by lobules (**b**). Each lobule (**c**) has a central vein in the middle and radially arranged cords of hepatocytes separated by sinusoids (radial purple lines) and bile canaliculi (radial green lines). The border of each lobule consists of loose connective tissue sheaths (red coating), which contain portal triads and interlobular vasculature branches in the corners of each lobule’s “hexagon.” As a result of DCL (**d**), all of the cellular elements are removed. (**e**) Detailed view of the sector of a single lobule. Each cord of hepatocytes contains two rows of the polarized cells. One side of the cell row is in contact with a sinusoid blood vessel (purple) through the space of Disse (yellow), while the other side of the cell row is in contact with a bile canaliculus (green) directly. Cells of the sinusoids and other cells of parenchyma, except hepatocytes, are not shown for simplicity. Blood from the portal vein (large blue blood vessel) and hepatic artery (smaller red blood vessel) is mixed in the sinusoids and transported through the liver parenchyma toward the central vein in the middle of the lobule. Bile is transported to the portal bile duct in the opposite direction. Portal vein, artery, and bile duct make a portal triad. (**f**) 3D view of the effect of DCL on the sector of a single lobule, shown at (**e**). Note the absence of cellular elements and preservation of collagenous stroma of interlobular septae and connective tissue sheaths of large blood vessels (red), basement membranes of large blood vessels and portal bile ducts (grey), and the ECM of Disse’s space (yellow). (**g**) 2D detailed schematic view of a segment of a lobule before (left) and after (right) DCL and segmentation of the parenchymal and stromal compartments. Note a portal triad (PT), containing a portal vein (PV), a hepatic artery (A), and an interlobular bile duct (D); sinusoids (S) with discontinuous endothelial lining separated from hepatocytes by Disse’s space (yellow). Narrow bile canaliculi (green) are visible between the rows of hepatocytes inside the cords. Bile canaliculi do not have their own cell linings. The central vein (CV) has the entire endothelial lining, basement membrane (BM), and collagenous sheath (red lines) around it. The structure of portal blood vessels is similar to that of the CV, but the layer of smooth muscle cells is thicker (it exists only before DCL). Red color indicates fibrous collagen. Black lines indicate basement membranes (BMs). Parenchymal compartment is shown by orange boxes, and the stromal one is indicated by blue boxes.

**Figure A5:**
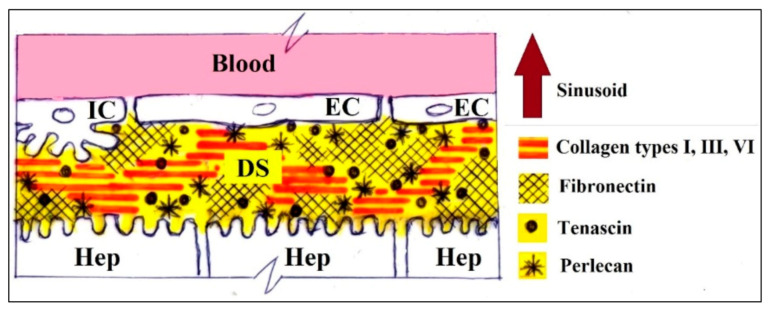
Schematic view of the relationship between sinusoids, Disse’s space, and hepatocytes (the parenchymal compartment of the liver). Note the wall of a sinusoid formed by discontinuous layer of endothelial cells (ECs) and absence of basement membrane underneath the ECs. The Disse’s space (DS; yellow) separates the endothelial cells of sinusoids from hepatocytes (Hep) and stellate cells of Ito (IC). The concentration of fibronectin increases along the sinusoids toward the central veins. Tenascin is relatively rare component. Perlecan is predominantly associated with basement membranes. Laminin is almost absent along the sinusoids in Disse’s space, except the area near the portal triads. The scheme is based on the information from the References [94,101,102,128].

The AOSSs were successfully prepared from the livers of CEs. The native structure of ED18 CE livers and the effects of the applied iWO-DCL procedure are detailed in Section A.3.1 and Section A.3.2 and are illustrated in Figure A3, Figure A4 and Figure A5 in Appendix A.3.1 and Appendix A.3.2. Briefly, iWO-DCL efficiently removed cellular elements, while the liver ECMs were well preserved (Figure 4) and structurally similar to the decellularized livers of other vertebrates [70,71,72].

DCL revealed *two histoanatomical compartments* in the LS-ECM that randomly appeared in the AOSSs (see Figure 4a,b). We termed the first compartment “parenchymal” and the second “stromal” for simplicity. The first compartment contained sponge-like structures corresponding to *the ECM of the former hepatic parenchyma* and formed mainly by the residuals of the Disse’s space elements. The second compartment was formed by denser structures relatively enriched with fibrillar collagen (see Figure 4b), mostly corresponding to *the former stromal elements* such as the walls of central veins, portal triads, and interlobular connective tissue sheaths and capsular connective tissue (see Figure A4 and Figure A5 in Appendix A.3.2). The relative area of the parenchymal and stromal compartments on the histological sections of LS-ECM (in the CE liver AOSS) was approximately equal (≈48 ± 21% and 52 ± 17%, respectively).

**Figure 5:**
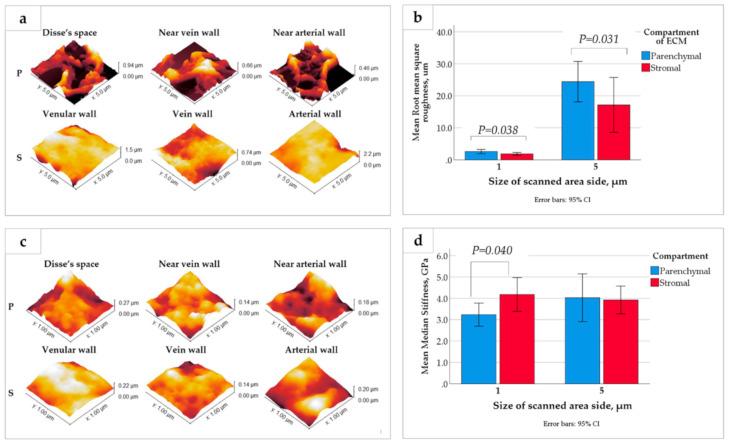
(**a**,**c**) Topography AFM images of the (**a**) micron scale, 5 × 5 µm, and the (**c**) submicron, 1 × 1 µm, areas of the dehydrated CE liver ECMs of parenchymal (P) and stromal (S) origin. (**b**,**d**) Quantitative analysis of the surface roughness (**b**) and stiffness (**d**) of dehydrated LS-ECM compartments. Notably, the graph in (**b**) shows that the LS-ECM compartments differ by root square roughness at both the micron and submicron scales (explored in 5 × 5 µm and 1 × 1 µm images, respectively) with statistical significance. At the same time, as shown in (**d**), the LS-ECM compartments differ by median stiffness with a statistical significance only at the submicron scale (as explored in 1 × 1 µm images).

**Figure A6:**
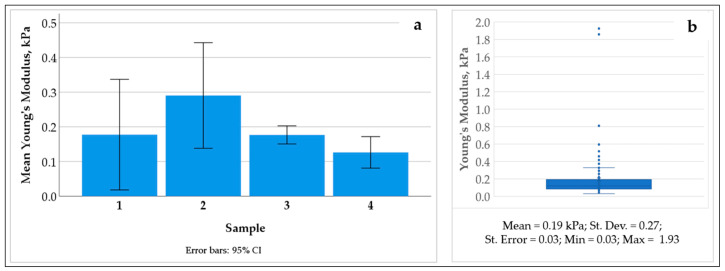
Mechanical characterization of the LS-ECM in hydrated state. Peak force mapping illustrates the distribution of the mean Young’s modulus across four samples of CE liver AOSSs (**a**) and overall distribution of the values in the whole set of samples (the dots show the individual values emerged beyond the 25%-75% interquartile range) (**b**).

**Figure A7:**
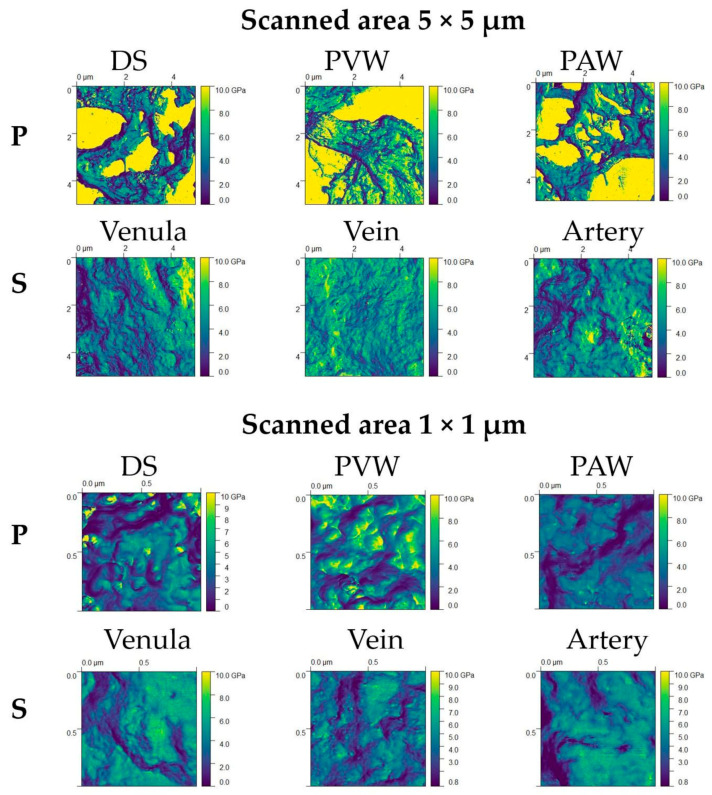
Stiffness mapping (normalized) of the different histoanatomical elements of the dehydrated parenchymal (P) and stromal (S) compartments of the LS-ECM in CE liver AOSSs measured on 5 × 5 and 1 × 1 µm AFM scans. Yellow tone codes higher stiffness values, deep blue tone codes the minimal stiffness (up to 2 GPa), and green tone codes stiffness in the range of 2–8 GPa. Note that the areas colored in yellow in the 5 × 5 µm scans of the parenchymal elements correspond to the areas without tissue (microscopy glass surface). The loose nature of the parenchymal ECM allows exposure of the glass surface, but results in very high variation of the stiffness values at the micron scale and masks any potential quantitative differences between the histoanatomical structures and compartments. The measurements on the 1 × 1 µm scans vary much less. Abbreviations: DS—Disse’s space; PVW—parenchymal ECM near vein wall; and PAW—parenchymal ECM near artery wall.

**Figure A8:**
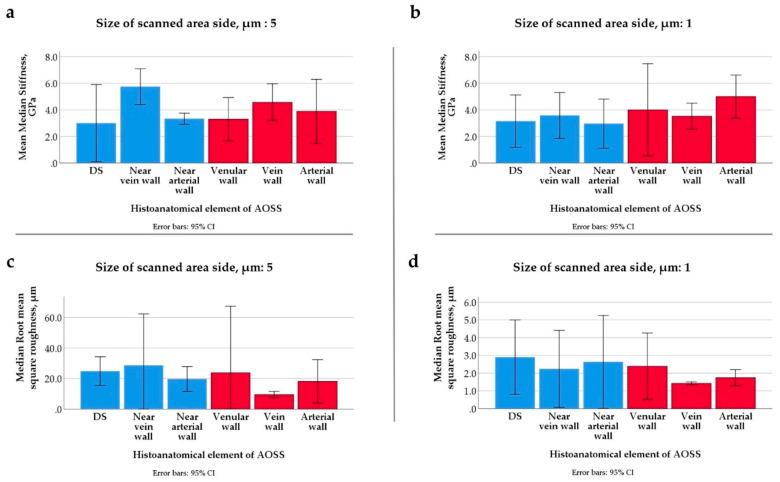
Physical properties of the histoanatomical elements of dehydrated LS-ECM (in CE liver AOSSs) measured in AFM scans with areas 5 × 5 µm (**a**,**c**) and 1 × 1 µm (**b**,**d**). The elements of the parenchymal compartment are shown by blue bars, and the elements of the stromal compartment are shown by red bars. (**a**,**b**) Median stiffness and (**c**,**d**) root mean square roughness. No statistically significant differences of stiffness and roughness were found between the studied histoanatomical elements at both AFM imaging scales (5 × 5 and 1 × 1 µm) in the given set of samples (*N* = 18 for each scanned area size). Abbreviations: DS—Disse’s space; “Near vein wall” and “Near arterial wall,” the properties of the areas of the parenchymal compartment measured in vicinity to veins and arteries, respectively.

The compartments of the LS-ECM were further examined using AFM (Figure 5 and Figure A6, Figure A7 and Figure A8 in Appendix A.3.3). The AFM analysis of the AOSSs in the hydrated state indicated that the Young’s modulus of the LS-ECM equals approximately 0.2 kPa (see Figure A6 in Appendix A.3.3). However, it was impossible to measure the mechanical characteristics of the scaffolds in wet form due to the very prominent surface topography. Therefore, we applied the AFM analysis of the dehydrated sections of the AOSS by the method proposed by us elsewhere [62].

Interestingly, the surface topography (see Figure 5a,c) and mechanical properties (see Figure 5b,d) of the dehydrated matrices of parenchymal and stromal origin varied depending on the scale of the measurements. The surfaces of the ECM derived from the CE liver parenchyma were highly uneven, in comparison to the more flattened stromal ECM. This feature was reflected by a statistically significant difference in the surface roughness of the compartments at both the micron and submicron scales (as measured in the 5 × 5 µm and 1 × 1 µm areas, respectively; see Figure 5b). At the same time, a statistically significant difference in the stiffness of the compartment-specific ECMs was observed only via the measurements in the smaller regions of interest (1 × 1 µm areas), while not obvious at a larger scale (see Figure 5d), which is due to the stronger influence of uneven surface topography in the 5 × 5 µm areas (see Figure A7 in Appendix A.3.3 for the details). The detailed exploration of the surface roughness and stiffness distribution across different histoanatomical elements of both compartments is shown in Figure A8 in Appendix A.3.3.

Meanwhile, below the level of statistical significance, there were notable features of the ECMs of vein walls and the parenchymal ECMs near the vein walls (see Figure A8 in Appendix A.3.3). The vein walls’ ECMs showed the lowest roughness among all of the studied histoanatomical structures at both the micron and submicron levels, and it was the stiffest one at the micron scale. The ECMs of parenchymal origin located near the vein walls had the highest stiffness and highest roughness in 5 × 5 µm samples (micron-scale), while at the submicron scale, the stiffest and the smoothest ones were among the ECM elements of parenchymal origin.

#### 3.2.2. TNBC Cells Differentially Colonize Parenchymal and Stromal LS-ECM, While ECMs Stimulate Phenotypic Plasticity of the Cells

**Figure 6:**
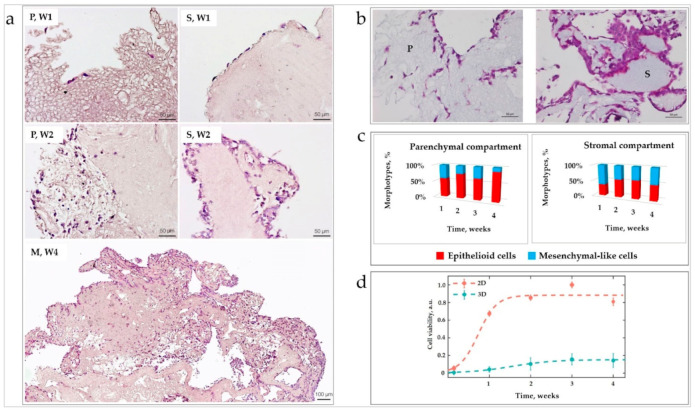
(**a**) Representative histological images of cellular colonization of the liver AOSSs during four weeks of in vitro culture (time points are labeled as W1, W2, and W4 for the first, second, and fourth weeks, respectively). The images of parenchymal and stromal compartments are labeled as “P” and “S,” respectively. “M” labels the remodeled ECM with mixed parenchymal and stromal features. Staining with H&E, the scale bars are 50 µm (top and middle rows) and 100 µm (bottom row). The top row shows the colonization patterns on the 5th day in vitro. Note sparse individual cells that are attached to the loose spongy-like parenchymal ECM on the outer border of the TEC (on the left). In contrast, formation of a discontinuous lining is visible on the dense ECM of the stromal compartment (on the right). Middle row, Day 13 in vitro: Note the colonization of the parenchymal compartment (P, W2) (the fragment with loose structure near the surface of AOSSs) by individual cells and predominantly single-cell invasion to the stromal ECM. A few small multicellular clusters showing predominant single-cell invasion in the deeper parts of the scaffold are visible on the border with the area composed by the stromal ECM. In parallel, in the stromal ECM compartment (S, W2): The continuous multi-row cell lining on the stromal ECM with minimal invasion is observable. Bottom row (M, W4): Remodeling of the ECM of stromal origin and massive diffuse colonization of the whole scaffold (day 28). (**b**) Parenchymal (P) and stromal (S) compartments of the CE liver ECM—TNBC 3D TECs on week 2. Staining by Masson’s trichrome method. Note similar tinctorial properties (light blue staining indicating comparable concentrations of collagen) and different distribution of the cells (stained purple) over the compartments. There are lower cell numbers and deeper invasion of individual cells in the parenchymal sector and formation of dense cellular clusters near the surfaces (with shallow invasion depth of cell collectives) in the stromal compartment. Scale bars, 50 μm. (**c**) Effect of parenchymal and stromal LS-ECM on cell morphology. Distribution of the epithelioid and mesenchymal-like cellular morphotypes in LS-ECM compartments during four weeks of in vitro culture in TECs. (**d**) Cell growth dynamics of MDA-MB-231 cells in 2D and 3D (TEC) in vitro cultures. Dashed curves represent the lines of best fit given by Equation (1).

**Figure A9:**
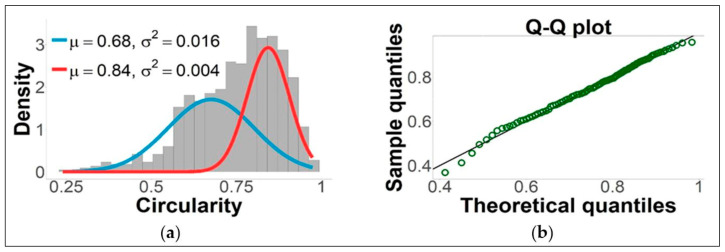
(**a**) A histogram of the circularity values of the total number of 706 individual cells examined in TECs collected after 1 week of in vitro culture. The data are best described by two normal distributions (blue and red curves represent probability density functions) and reveal the formation of two subpopulations of cancer cells with different circularities such as elongated oval-shaped “mesenchymal-like” and nearly circular-shaped “epithelioid” cells with circularity values of ~0.68 and ~0.84, respectively. (**b**) Comparison between the quantiles of the theoretical bimodal normal distribution with the quantiles of the observed data. The data points are positioned close to y = x, indicating that the distribution of the sample data is similar to the normal distribution.

The distinctive patterns of initial cellular attachment and subsequent colonization in the parenchymal and stromal LS-ECM compartments were observed in the liver-TNBC TECs cultured over four weeks in vitro (Figure 6a,b). Following the contact with parenchymal and stromal LS-ECM for five to seven days, the MDA-MB-231 cells formed two subpopulations with different cell circularities, as shown in Figure A9 in Appendix A.3.3. These subpopulations are further referred to as “epithelioid” (more circular) and “mesenchymal-like” (elongated) morphotypes; the ratio of the morphotypes across the LS-ECM compartments changed with time (Figure 6c). The overall cellularity of the different compartments of the TNBC-liver TECs is shown in Table A2 in Appendix A.3.3. The time course of the colonization of the liver AOSS by TNBC cells is described below.

During Week 1 (see Figure 6a, top row), the number of cells attached to the ECM of the stromal compartment was 2.8 times higher than to the parenchymal ECM. Notably, at this time point, most cells in the in the stromal compartment formed single-row linings at the outer surfaces of the ECM, while the cells in the parenchymal compartment were mainly distanced from one another. In the parenchymal compartment, a slight majority of cells had epithelioid morphology (59% ± 16%), and the rest appeared mesenchymal-like. More than a half (63% ± 10%) of the cells observed in the stromal compartment had a mesenchymal-like elongated appearance (see Figure 6c).

At the end of Week 2 (see Figure 6a, middle row, and Figure 6b), in the parenchymal compartment, diffuse individual-cell colonization was predominantly (75% ± 23%) observed, while the remaining cells formed small clusters and single-cell linings over the AOSS surface. The individual cells mainly had epithelioid morphology (76% ± 7%), and the rest were classified as mesenchymal-like. In the stromal compartment, cells mostly aggregated in multicellular clusters, both on the scaffold surfaces (as double- or triple-row linings or islands) and in the depth of the matrix. Only 17% ± 21% of cells in the stromal compartment remained single. The stromal ECM was mostly invaded by groups of cells. The invasion occurred primarily along the voids and clefts in the scaffolds. These clusters invaded the stromal parts of AOSSs to a depth (defined as a distance from the AOSS surface) of 70 ± 24 µm. In the parenchymal compartment, the individual cell invasion prevailed and reached the deeper parts of the scaffolds (134 ± 54 µm). On weeks 2 to 3, a tissue-like structure of the micrometastatic tumor formed.

During weeks 3 and 4 (see Figure 6a, bottom row), a notable sponge-like remodeling of the ECM was observed in the stromal compartment, resulting in a blurred distinction between the ECM of the parenchymal and stromal origins. The invasion of cancer cells progressed over this period, reaching up to an 800 µm depth from the AOSS surface regardless of the matrix origin. By the end of week 4, approximately 70% ± 16% of single cells in the TECs had epithelioid morphology.

Analysis of the distribution of the individual cells with epithelioid and mesenchymal-like morphotypes across parenchymal and stromal compartments during four weeks of in vitro culture is shown in Figure 6c. A statistically significant dominance of mesenchymal-like shaped cells (possibly reflecting epithelial-to-mesenchymal transition, EMT) was observed in the stromal compartment on week 1. A statistically significant dominance of the epithelioid cell shape (possibly reflecting mesenchymal-to-epithelial transition, MET) was observed in the parenchymal compartment on the weeks 2 and 4, and in the mixed compartment (not shown in the figures) on week 4.

#### 3.2.3. Cell Growth Dynamics in 3D TECs Is Different vs. 2D Cultures

A comparative quantitative analysis of MDA-MB-231 cell growth in 2D monolayer cultures and 3D liver TNBC TECs was carried out using an MTT assay (Figure 6d). The amount of viable cells that successfully attached to the surface of AOSSs on day 1 was found to be ~15%–20% of the matching 2D culture seeded in plastic culture plates. This ratio corresponds to the ratio of the external surface area of the AOSSs (~0.4–0.8 cm^2^) to the area of a well in a 24-well plate (~2 cm^2^), indicating that the attachment efficiency in 3D TECs was approximately the same as on cell culture-treated plastic and confirming high contact biocompatibility of the CE liver scaffolds.

The cells in 2D cultures and 3D TECs presented strikingly different growth behavior. As is shown in Figure 6d, the cell density increased until day 21 in both cultures, but with different rates. During week 1*,* in the 2D cultures, the growth was much faster than in the TEC counterparts. In the following two weeks, the weekly cell counts increased similarly in the 2D cultures and 3D TECs due to the deceleration of the growth rate in monolayers and the slow but steady proliferation of cells in TECs. Importantly, the cell growth in TECs was stabilized between weeks 2 and 3, indicating the most reliable and reproducible phase of the 3D culture model with high viability and predictable numbers of the cells per construct, which is needed for drug and nanoparticle testing and many other potential applications. As discussed above, at this stage, TECs also acquired the tissue-like structure of the reconstructed micrometastasis.

In the final week, week 4, the growth rates in both types of cultures decreased in comparison to that of week 3. This decrease was less pronounced in the TECs. Between days 21 and 28 in vitro, the cell viability in 2D cultures decreased by approximately 19% ± 10%, and in the TECs by 8% ±36%. Differences in the viability of cells between 2D and TEC cultures were statistically significant at each time point (*p* < 0.001).

The observed cell population dynamics in the matching 2D and 3D in vitro cultures of MDA-MB-231 cells closely followed a general logistic growth model (see Equation (1) in Materials and Methods). The cell population growth rate (*d*) in 3D TECs was lower compared to the growth rate in the 2D cell culture (*d* = 0.28 days^−1^ in 3D vs. *d* = 0.63 days^−1^ in 2D). The results of the fitting and the estimated values of the model parameters are provided in Table A3 and Table A4 (Appendix A.3.3), respectively.

**Figure A10:**
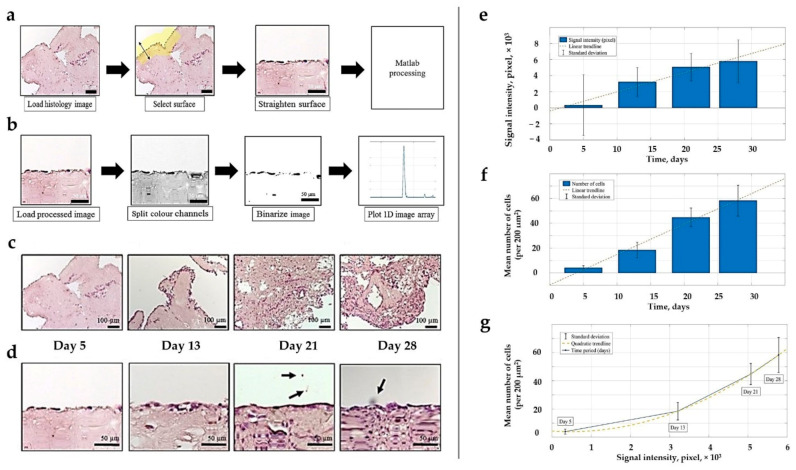
(**a**,**b**) The procedure of the digital image analysis of surface colonization pattern illustrated with a histology image of the CE liver/TNBC TECs at day 5 of the in vitro culture. (**a**) The first part of the algorithm is implemented in ImageJ and includes loading of image, selection of the surface area of 300 pixels in width, and straightening of the selected surface. The arrow on the yellow shade indicates a width of 300 pixels. (**b**) The second part of the algorithm is implemented in MATLAB and includes loading of the image processed in ImageJ, splitting of color channels, binarization of the green channel image with the with a threshold of 0.35, and creation of 1D image array, e.g., the image’s signal intensity is plotted versus the matrix depth in micrometers. (**c**,**d**) Distribution of the cells near the surface of the TECs over a period of 28 days. (**c**) Original histology images and (**d**) corresponding images of the digitally straightened and analyzed surface areas of the TECs. Arrows indicate sample impurities, which may occur during sample preparation. (**e**–**g**) Results of the digital image analysis of the TECs’ surfaces colonization patterns. Note the linear growth of cellular density near the TECs surfaces over the observation time. (**e**) Signal intensity in the surface areas of the TECs over the time (28 days). (**f**) Average number of cells per 200 µm^2^ in the surface areas of the TECs over time (28 days). (**g**) Average number of cells versus signal intensity (pixel) at four different time points (5, 13, 21, and 28 days). Scale bars: (**a**) left and middle panels 100 µm, and the right panel 50 µm; (**b**) and (**d**) all panels 50 µm; (**c**) all panels 100 µm.

Interestingly, while the overall cellular growth in the TECs (estimated using the biochemical MTT assay) followed the logistic model, the subpopulation of the cells localizing near the scaffolds’ surfaces (within the outer 300 µm of the ECM), as measured via digital image analysis, behaved differently and grew linearly with time (Figure A10 in Appendix A.3.3).

#### 3.2.4. 3D TECs Are Angiogenic In Vivo

**Figure 7:**
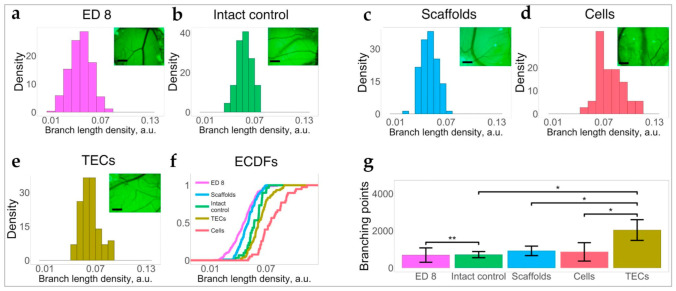
The angiogenic effects of 3D TECs, liver AOSSs (scaffolds), and suspensions of MDA-MB-231 cells (cells), grafted on a chick embryo CAMs compared to natural growth of CAM vasculature (intact control) in the period between ED8 and ED12 of the embryonic development. Histograms of branch length density of (**a**) CAM vasculature on the day of grafting (ED8), and in (**b**) intact control CAMs (ED12), (**c**) scaffolds, (**d**) cells, and (**e**) TECs. The insets are images of blood vessels located in the vicinity of grafted materials. Scale bars, 2 mm. (**f**) ECDFs of branch length density in CAMs on ED8 and in each group on ED12. (**g**) Comparison of the number of branching points (per unit area) in CAM blood vessels in the studied groups. Error bars indicate standard deviation. Stars denote the level of statistical significance by Mann–Whitney test: * *p* < 0.05 and ** *p* < 0.01.

**Figure A11:**
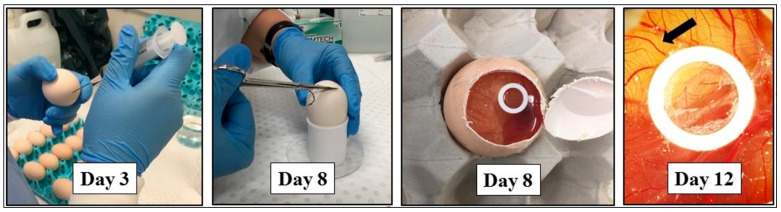
Illustration of the grafting protocol. The following sequential steps are shown: Extraction of the egg white on ED3. Opening of the egg on ED8. Grafting of the tested samples on the CAM on ED8. The cell suspension grafting is shown; a sterile white rubber ring taken from a 1.5 mL cryovial (inner diameter 8 mm) is used to prevent the leakage of the suspension. The last (right) image shows the same egg on ED12. The arrow shows a bifurcation of a blood vessel as a preferential grafting site.

**Figure A12:**
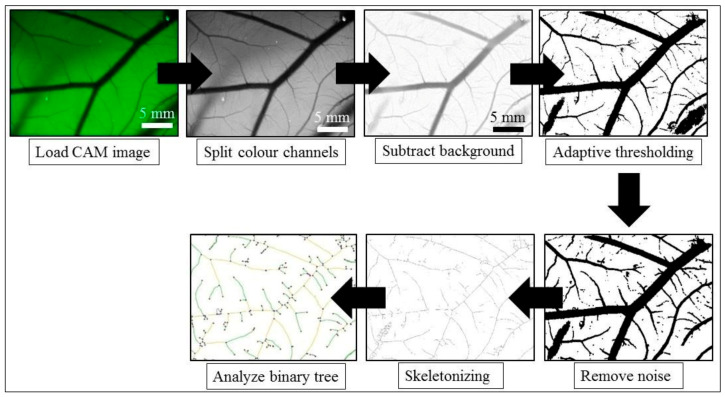
Algorithm used for the validation of induced blood vessel growth. Angiogenesis Analyzer was applied for the skeletonizing and analysis of the obtained binary tree.

**Figure A13:**
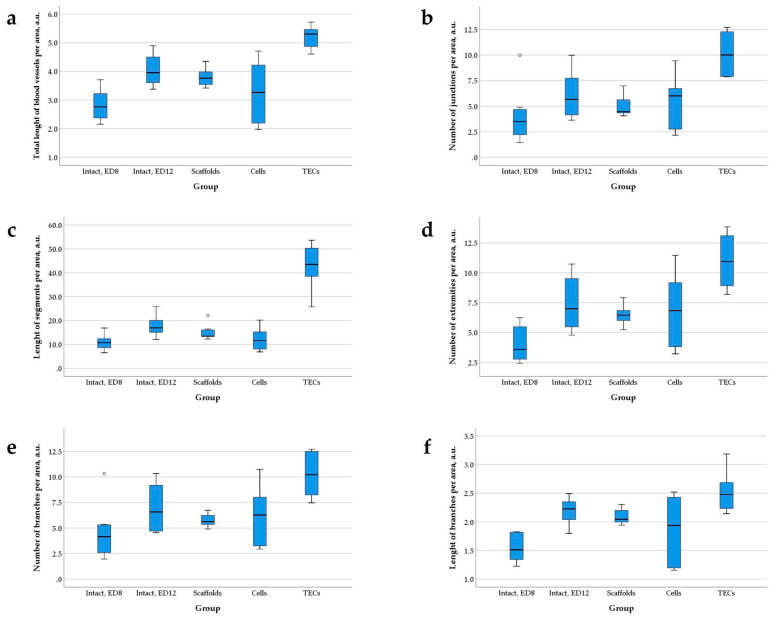
Quantitative analysis of angiogenesis. The parameters of the blood vessel tree are reflected as values per unit area. (**a**) Total blood vessels’ density, × 10^−5^; (**b**) number of Junctions, × 10^−10^; (**c**) length of segments, × 10^−5^; (**d**) number of extremities, × 10^−10^; (**e**) number of branches, × 10^−10^; (**f**) length of branches, × 10^−5^. The results are presented as boxplots, where thick dark horizontal line shows mean values, blue boxes indicate CI_95%_ for the mean, and the whiskers indicate the minimal and maximal observed values.

**Figure A14:**
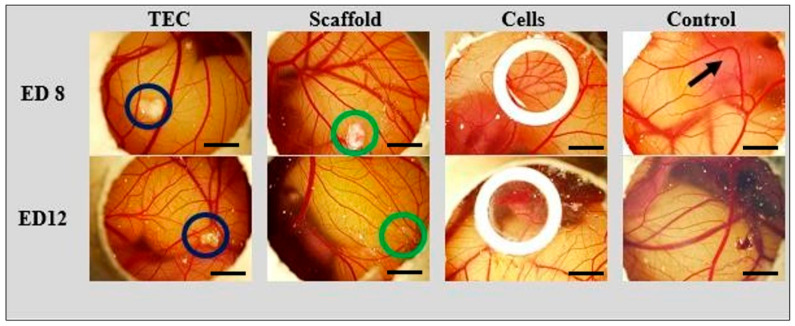
Architecture of CAM blood vessels in studied groups. Low-magnification (0.63×) view of the control and grafted CAMs on ED8 and ED12 obtained using bright-field microscopy. Angiogenic effects induced by the following chick embryo CAM grafts: 3D TEC (TEC; labeled with blue circles), liver AOSSs (scaffolds; labelled with green circles), and suspension of MDA-MB-231 cells (cells; xenograft suspension was placed into the chamber formed by a white rubber ring appeared on the photos as white circles), in comparison to natural embryonic development of CAM vasculature (control) during a period between ED8 (the area matching the grafting zone in the experimental groups is shown by the arrow) and ED12. Note that the degree of vascularization of CAM on ED8 was naturally variable between embryos. Representative images of the same eggs on ED8 and ED12 were used for analysis and shown for each group. Equal volume samples of the grafted TECs and AOSSs were used. The number of cells (2 × 10^5^) in the cellular xenograft and TECs were approximately equal. An arrow in the control CAM, ED8, indicates the chick embryo residing underneath the CAM. The white dust on the surface of CAM is small fragments of the eggshell dropped on the CAM during the shell windowing. Note blood vessels converging toward the grafts in the TEC and scaffolds groups. Scale bars, 5 mm.

The angiogenic potential of the TNBC liver TECs and CE liver AOSSs in vivo was examined using the chick embryo CAM assay, as described in Appendix A.3.4, Appendix A.3.4.2, and shown in Figure A11, Figure A12, Figure A13 and Figure A14. We found that grafting of CAMs with 3D TECs, CE liver AOSSs, and suspensions of MDA-MB-231 cells induced different changes in natural angiogenesis in CAMs occurring between ED8 and ED12 and resulting in specific architecture of blood vessel trees in the studied groups (Figure 7 and Figure A13 in Appendix A.3.4). The convergence on the host blood vessels toward the graft was clearly visible in CAMs grafted with TECs, but it was less obvious in other groups (see Figure A14 in Appendix A.3.4).

The TECs and cell xenografts induced a statistically significant increase in branch length density in CAM blood vessels, in comparison to the intact control, while grafting of the AOSSs did not result in this angiogenic switch (Figure 7a–f and Figure A14 in Appendix A.3.4). In contrast to other types of xenografts, the TECs also induced intensive branching of blood vessels (Figure 7g), resulting in increased total blood vessel density (see Figure A13a in Appendix A.3.4), accompanied by the formation of long torturous segments between the branching points (see Figure A13c). At the same time, the elongation of the branching blood vessels in CAMs grafted with TECs and scaffolds was rather inhibited as demonstrated in Figure 7c,e. The combination of these changes led to the formation of the areas of denser blood vessels networks between the proper branching points. Finally, as shown in Figure A13, in TEC-grafted CAMs, the total blood vessel density, and the segment length per area were increased in comparison to all other groups.

Figure 7f overviews the empirical cumulative distribution functions (ECDFs) visualizing the branch length density distributions in intact CAMs on ED8 and in each of the studied groups on ED12, and the summarized data presented in Figure 7a–e. These results show that compared to the measurements on ED8, the CAMs that developed naturally underwent detectable angiogenesis, while the CE liver AOSS did not enhance this at the level of statistical significance. At the same time, the TECs supported the development of higher branch length densities in CAMs than that occurred in the naturally developing embryos and in CAMs grafted with the unseeded scaffolds (Figure 7g). The most pronounced increase in the branch length density was detected in the cell group.

### 3.3. The 3D TNBC-Liver TECs as Testbeds for Drug and Nanoparticles Testing

#### 3.3.1. TNBC Cells in 3D TECs Are Less Sensitive to the Cytotoxic Action of Dox and AMS-6-Dox Than in 2D Cultures of MDA-MB-231 Cells

The cytotoxic effect and cellular uptake of the chemotherapeutic drug doxorubicin (Dox) were evaluated in parallel in 3D TECs and 2D monolayer cultures of MDA-MB-231 cells. Dox was applied to 3D and 2D models of TNBC in equal concentrations. It was delivered to the cells either in free (molecular) form or by anionic surfactant mesoporous silica nanoparticles loaded with Dox (AMS-6-Dox).

**Figure 8:**
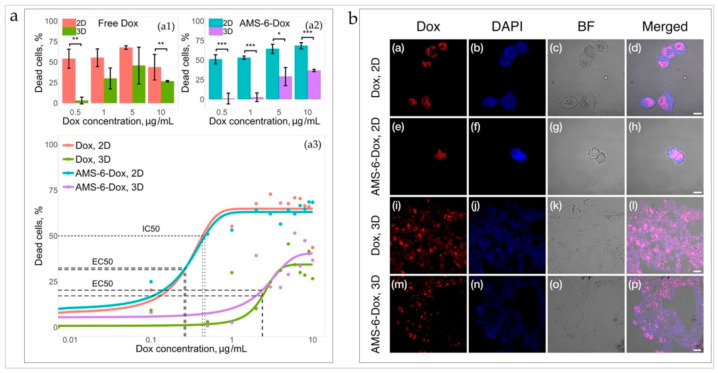
(**a**) Cytotoxicity of free and nanoformulated Dox in 2D cultures of MDA-MB-231 cells and 3D TECs. The top graphs: The effect of free Dox (on the left) and the effect of AMS-6-Dox (right graph). Error bars indicate CI_95%_; statistically significant difference at * *p* < 0.05, ** *p* < 0.01, *** *p* < 0.001 by Mann–Whitney *U*-test. Bottom graph: Dose–response curves of free Dox and AMS-6-Dox in 2D and 3D in vitro cultures. Solid lines represent the lines of best fit given by Equation (3) (see Table A6 and Table A7 in Appendix A.3.5.2 for fit parameters). Note that in the 3D culture, the IC_50_ could not be calculated within the studied concentration range (up to 10 µg/mL). (**b**) Confocal microscopy images of MDA-MB-231 cells incubated for 24 h with (**the upper row**) Dox in 2D; (**the second row**) AMS-6-Dox in 2D; (**the third row**) Dox in 3D; and (**the fourth row**) AMS-6-Dox in 3D TECs. Intrinsic Dox fluorescence was detected in the red channel (Dox), while DAPI fluorescence (blue channel, DAPI) was used for contrasting of cell nuclei. Control bright field (BF) images were acquired to visualize the tissue structures, and merged images highlight the colocalization of Dox and DAPI signals. Dox concentration, 10 μg/mL. Scale bars, 10 μm (Dox, 2D; AMS-6-Dox, 2D) and 20 μm (Dox, 3D; AMS-6-Dox, 3D). The experiments were repeated twice, with at least triplicates for each condition. The illumination conditions were kept constant for every imaging channel.

**Figure A15:**
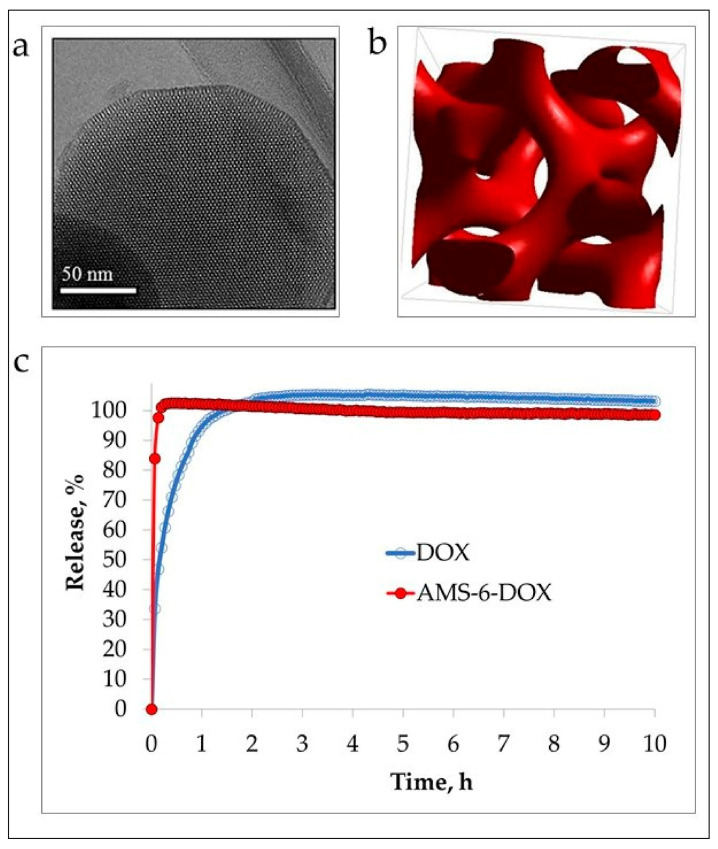
(**a**) Typical TEM images of calcined AMS-6 mesoporous material showing a high degree of mesoporous order and (**b**) schematics of the of AMS-6 structure. (**c**) Kinetics of Dox release from AMS-6-Dox vs. the free Dox drug dissolution in PBS.

**Figure A16:**
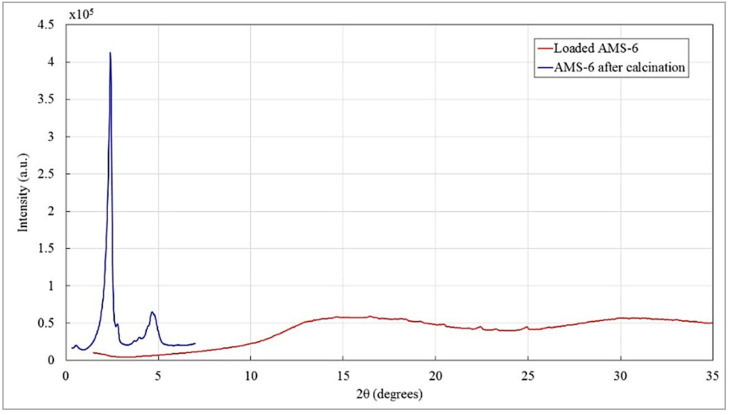
X-ray diffraction (XRD) pattern of pure calcined AMS-6 mesoporous nanoparticles (blue) and the loaded AMS-6-Dox nanoparticles (red). The measurements of the pure calcined AMS-6 NPs reveal a highly ordered mesostructured with peaks of scatter in-between the pores of the nanoparticles. The typical XRD pattern of the pure sample display peaks at low angles of 2.4° and 4.8° with an intensity of 4.12 × 10^5^ and 0.6 × 10^5^ a.u., respectively. The loaded AMS-6-Dox sample curve shows small peaks at high angles of approximately 15° and 30° with intensities below 0.75 × 10^5^ a.u., which are most likely the scattering patterns of crystallized Dox outside of the pores. The pores of the AMS-6-Dox still seem to be filled with DOX, since the pattern does not show any peaks at low angles, which would indicate scatterings between the pore walls of the nanoparticles.

**Figure A17:**
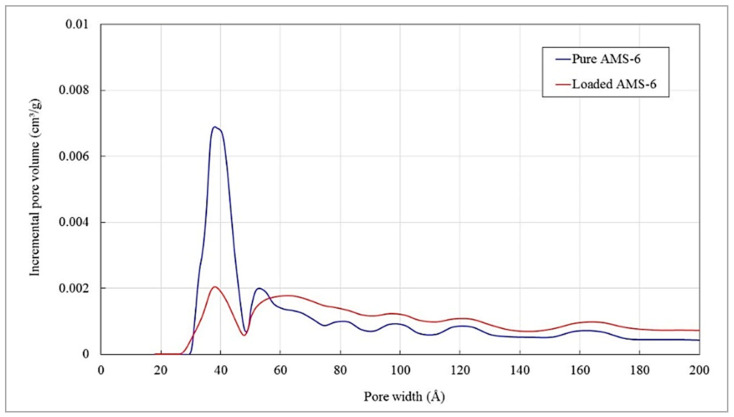
DFT pore size analysis. The pore width of pure AMS-6 and loaded AMS-6-Dox nanoparticles is in the range of 30–50 Å.

**Figure A18:**
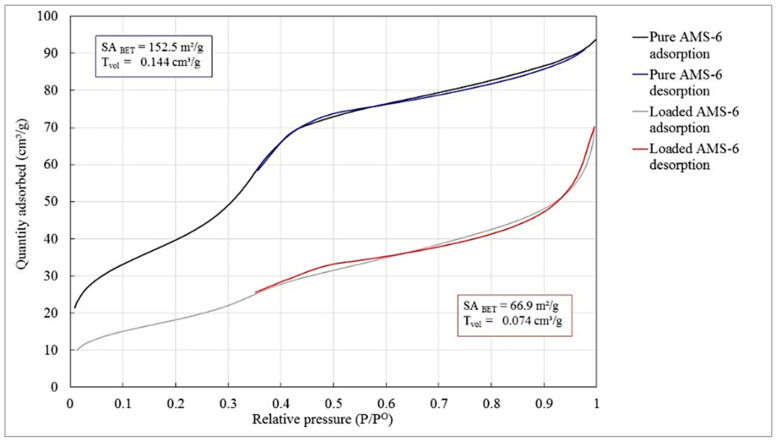
Nitrogen adsorption/desorption isotherm curves of pure AMS-6 and loaded AMS-6-Dox nanoparticles. The very narrow hysteresis loop where the plotted adsorption and desorption curves are close together results from the fact that the pore width of 3.81 nm is very small.

**Figure A19:**
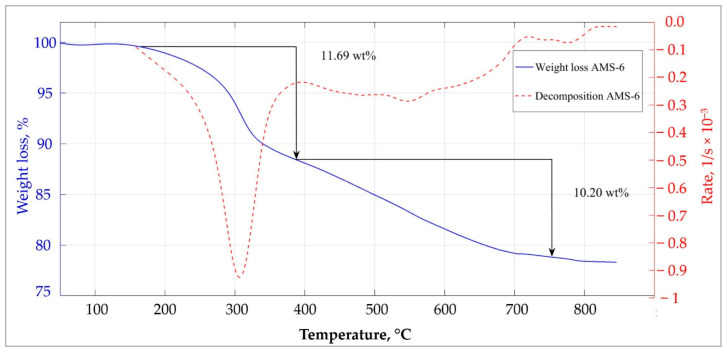
Thermogravimetric analysis of the unloaded AMS-6 nanoparticles sample. The thick blue line corresponds to the weight loss versus the temperature, and the dashed red line represents the first derivative from the thermogravimetric analysis (DTG) curve. The latter shows characteristic decomposition peaks, e.g., at 380 °C. The measurement was performed on the pure AMS-6 to confirm the absence of any organic compounds that could block the pores, which were supposed to contain the loaded drug later. The peak of DTG at approximately 310 °C corresponds to the loss (decomposition) of propyl amine functionalized groups. The analysis also revealed the incorporation of approximately 11.69 wt% of covalently bound propyl amine groups in the pores of the NPs. However, with 11.69 wt%, the presence of amine groups was slightly elevated. A higher value would indicate a high amount of remaining amino groups, which would mean a blockage of the NPs pores. The remaining weight loss above 400 °C was due to evaporating water (also known as extra framework water) caused by condensation of silanol groups.

**Figure A20:**
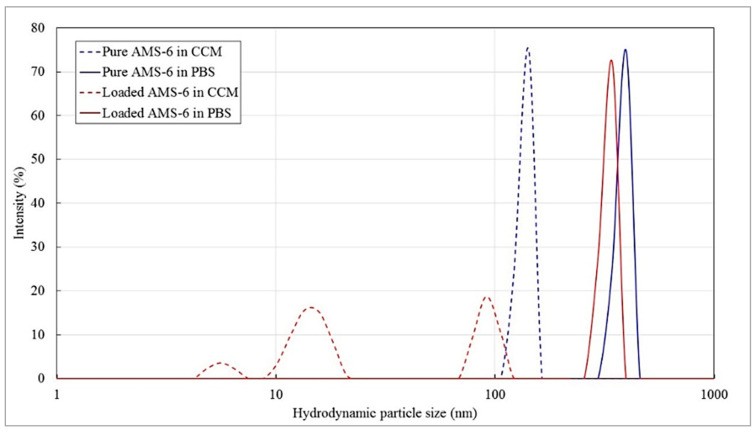
Hydrodynamic size distribution of pure and loaded AMS-6 NPs in complete culture medium (CCM) and in PBS. Note the variation of the particles’ size distribution depending on the used dilution medium.

**Figure A21:**
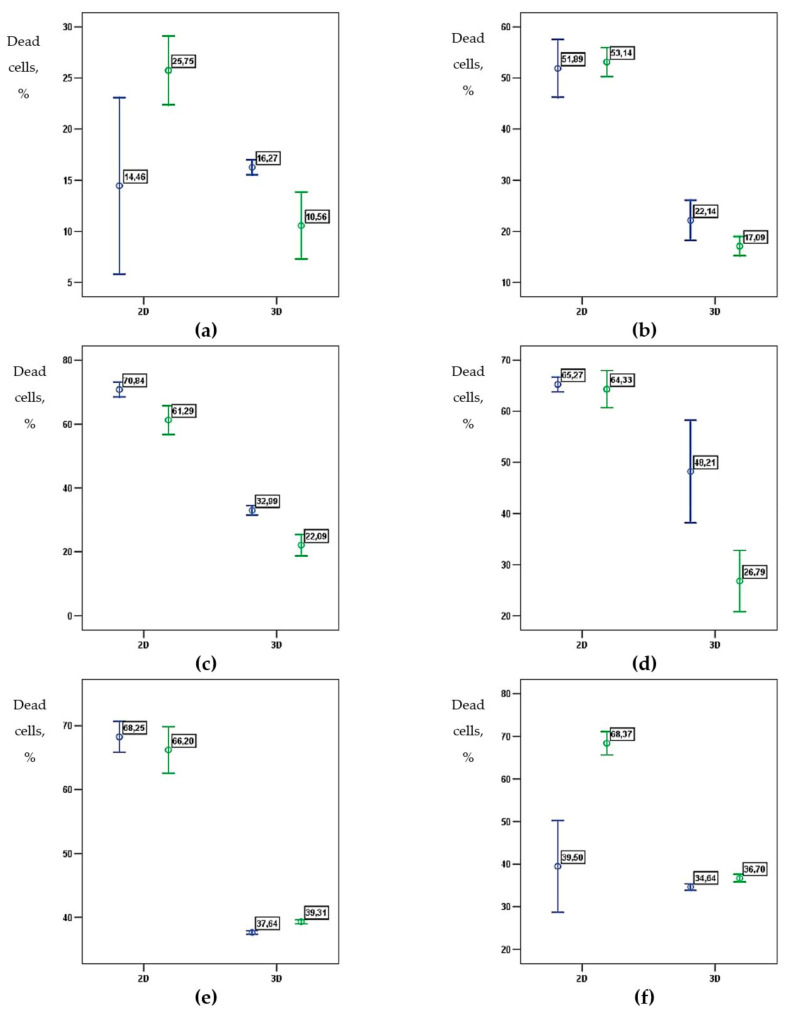
Intergroup comparisons of the cytotoxic effect of free and nanoformulated Dox in the 2D culture of MDA-MB-231 cells and 3D TECs after 36 h exposure. The mean ratio of dead cells after application of free Dox (blue) and AMS-6-Dox (green), respectively, is shown for the following concentrations of Dox: (**a**) 0.1 µg/mL; (**b**) 1 µg/mL; (**c**) 3 µg/mL; (**d**) 5 µg/mL; (**e**) 7 µg/mL; and (**f**) 10 µg/mL. Error bars show 95% confidential intervals for the mean percent of dead cells. Data labels on the graphs also indicate the mean percentage of dead cells in the group.

AMS-6-Dox was prepared and carefully characterized, as described in the Materials and Methods, and the results are shown in Figure A15, Figure A16, Figure A17, Figure A18, Figure A19, Figure A20 and Table A5 in Appendix A.3.5.1. The results of MTT viability assays performed in 2D and 3D TEC cultures incubated for 36 h in the presence of molecular and nanoformulated Dox are shown in Figure 8 and in Figure A21 (Appendix A.3.5.2). In a control study, we found that pristine AMS-6 nanoparticles were not cytotoxic in both 3D TECs and 2D cultures at a concentration range 0–250 µg/mL, which is consistent with our previous studies [73].

The cells in 3D TECs appeared much more resilient to both molecular and nanoformulated Dox in comparison to cells in 2D cultures (Figure 8a, top graph). The dose–response relationship in each group was described, using a sigmoid function (see Materials and Methods for the details; Equation (3)). A half-maximum effective concentration, EC_50_, in 3D TECs was found to be approximately 9 times higher than in 2D cultures for both formulations (see Table A6 and Table A7 in Appendix A.3.5.2). Moreover, in the 2D culture, the half-maximum inhibitory concentration, IC_50_, was found to be 0.43 µg/mL and 0.46 µg/mL for free and nanoformulated Dox, respectively, while in 3D TECs, the 50% cell death threshold was not reached, indicating a markedly worse efficacy of both formulations in TECs compared to conventional 2D cultures (Figure 8a, bottom graph).

The data also show the effect of Dox delivery formulation (free or nanoformulated) on the cellular response in both 2D and TEC cultures (see Figure 8a and Figure A21 in Appendix A.3.5.2). Nanoformulated Dox was generally more cytotoxic at higher Dox concentrations (>5 µg/mL), while the efficacy of free Dox was reduced at the higher Dox concentrations in both 2D and 3D cultures. At a maximum studied Dox concentration of 10 µg/mL the difference in mean viability between 3D and 2D cultures was 17.1% and 31.7% for Dox and AMS-6-Dox, respectively (see Figure 8a).

#### 3.3.2. Uptake of Dox and AMS-6-Dox in 3D TECs and 2D Cultures of MDA-MB-231 Cells

**Figure A22:**
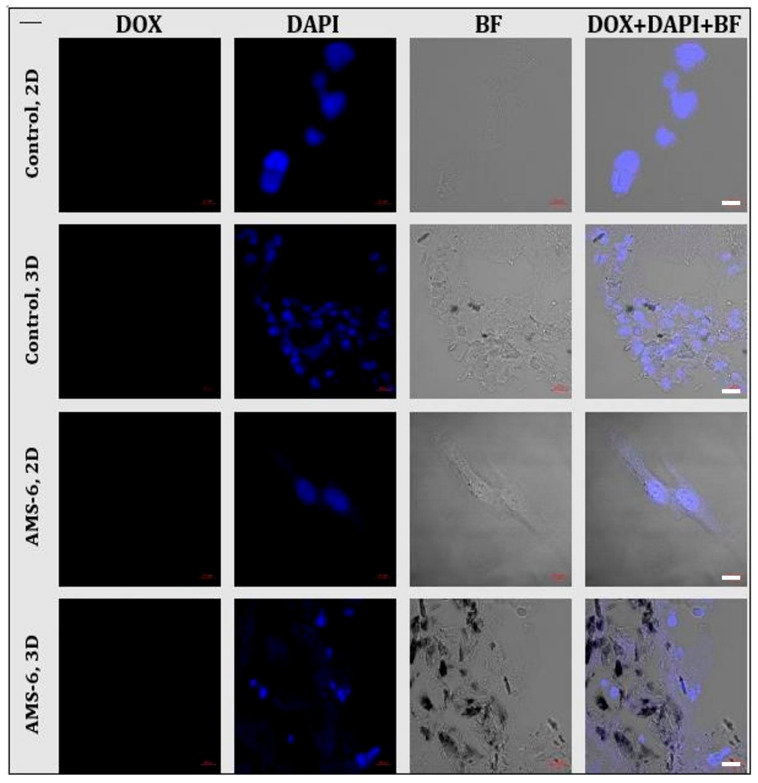
Laser-scanning confocal fluorescence microscopy images 2D and 3D TECs in vitro cultures of MDA-MB-231 cells incubated with complete culture medium (control 2D and 3D) and pure (not loaded with Dox) AMS-6 nanoparticles for 24 h. Note the absence of Dox fluorescence in the red channel (Dox), the clear association of DAPI fluorescence staining with cell nuclei (blue channel, DAPI). AMS-6 concentration, 50 μg/mL. Scale bars: Control and AMS-6, 2D: 10 µm; Control and AMS-6, 3D: 20 µm.

**Figure A23:**
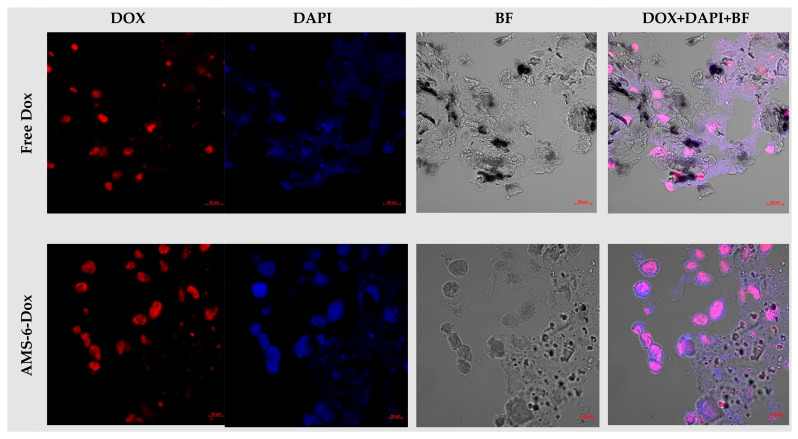
Laser-scanning confocal fluorescence microscopy images in 3D TECs, combining CE LS-ECM and human MDA-MB-231 cells that were incubated with free Dox and AMS-6-Dox nanoparticles for 24 h. Intrinsic Dox fluorescence was detected in the red channel (Dox), while DAPI fluorescence (blue channel, DAPI) was used for contrasting of cell nuclei. Control bright field (BF) images were acquired to visualize the tissue structures, and merged images highlight the colocalization of Dox and DAPI signals. Note the absence of preserved cell nuclei in the depth of TEC treated with AMS-6-Dox nanoparticles (the bottom row), in comparison with TECs treated with free Dox (upper row). Dox concentration, 10 μg/mL. Scale bars, 20 μm (upper row) and 10 μm (bottom row). The illumination conditions were kept constant for every imaging channel.

The results of the evaluation of cell and tissue uptake of Dox and AMS-6-Dox in 2D and 3D TEC cultures are shown in Figure 8b, Figure A22, Figure A23 (Appendix A.3.5.2). In the 2D cultures, the fluorescent signals of free Dox and AMD-6-Dox nanoparticles were visible in the cellular nuclei (Figure 8b (the upper two rows)). In contrast, in the 3D TECs, fluorescence signals of free and nanoformulated Dox were distributed between the cell nuclei, cell cytoplasm, and ECMs of TECs. Free Dox was noticeably accumulated not only in the cell nuclei, but also throughout the TECs’ ECMs, while fluorescence from AMS-6-Dox in 3D TECs was mainly detected in the nuclei of cells located at the TEC surface and only diffused DAPI staining and Dox-positive cell debris was observed at a depth of >50 µm in TECs, indicating destroyed nuclei and destroyed tumor cells, respectively (Figure 8b (the lower two rows)).

To sum up, the MTT test and imaging results show that the distribution and cytotoxic effects of free and nanoformulated Dox were influenced by the liver-specific ECM microenvironment, and the method of Dox delivery to 3D TECs modulated its local therapeutic activity.

## 4. Discussion

### 4.1. Methodological Advancements

Our study shows that the CE tissue-engineering platform for modeling cancer micrometastases is feasible, reproducible, and affordable. Fertilized eggs can be obtained from industry poultry suppliers, implying low-cost, well-controlled, and infection-free production [74]. We estimated that 100–150 LS-ECM scaffolds could be prepared from a single liver of an ED18 broiler CE, with the resulting cost of 0.01–0.02 USD per scaffold. The cost of CE AOSSs derived from the other organs may be higher due to the lower volume and/or ECM content. The considerable price reduction compared to animal research incentivizes the application of CE tissue engineering products in various areas of research and development. Our proposed iWO-DCL procedure avoids the complexities of perfusion-based WO-DCL, while providing whole-organ acellular scaffolds with high biocompatibility and a long shelf life. The presented model allows cultivating the 3D tumors in vitro for long periods (at least one month, as demonstrated in this work), which is important for metastatic cancer research. Earlier, we applied the iWO-DCL protocol to food-grade livers of mature chickens and successfully created biocompatible AOSSs and TECs [75]. However, the available organ types are limited among food-grade materials, while their quality and sterility may vary.

The 3D TECs may also be transferred to in vivo settings as in vitro pre-cultured controllable tumor xenografts, offering a unique possibility to observe intrinsic mechanisms behind metastatic cancer outside and inside the body. Notably, the panels of AOSSs can be prepared from the organs of genetically related animals (CEs) without using the specialized vivarium setups and procedures. Furthermore, the immaturity of the immune system and reduced pain sensitivity of the CEs, as well as the easiness of manipulations on the extraembryonic membranes [76,77], makes CEs a promising experimental system for grafting of the 3D TECs on CAMs, as demonstrated in our study. Taken together, the 3D tissue engineering modeling of tumors in the CE platform is an attractive option for high-throughput research and pharmaceutical applications at the in vitro/in vivo interface.

### 4.2. Cancer Biology Findings

#### 4.2.1. Organ-Specific ECMs Differentially Modulate TNBC Cell Attachment and Growth

Using the multiorgan panel of CE AOSSs, we explored the role of organ-specific ECMs in metastatic organotropism. Our tumor of interest was TNBC. TNBC is an aggressive HER2-, estrogen, and progesterone receptor-negative mammary carcinoma [78,79,80]. A lack of targeted therapy and limited efficiency of conventional treatment contribute to the low survival of the patients with metastatic TNBC [81]. Among many subtypes of breast cancer, TNBC has one of the highest propensities to distant metastases [82,83], especially to the visceral organs [15] and abdominal cavity [82]. The most common places of the distant metastases of TNBC are the lungs (31%), bone (27%), liver (15%), central nervous system (15%), and pleura (12%) [84].

**Figure A24:**
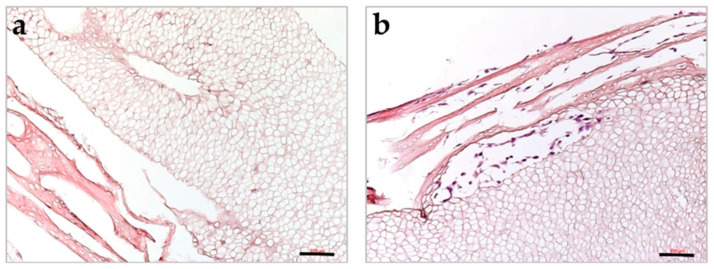
(**a**) Effect of iWO-DCL with an additional decalcination stage on CE tibial bone. (**b**) Recellularization of the bone AOSS with MDA-MB-231 cells. Staining with H&E, scale bars 100 µm.

MDA-MB-231 cells are invasive TNBC cells that possess an EMT-associated mesenchymal-like phenotype in vitro [85]. In our study, these cells demonstrated the attachment and colonization “preference” of the organ-specific ECM (see Figure 3) similar to the clinically observed TNBC organotropism [82,84,86]. The lung ECMs were the most hospitable substrate for TNBC cells, while the ECMs of the heart and brain were the least preferred, as indicated by a statistically significant reduced cellularity. In the liver ECMs (LS-ECMs), the colonization efficiency was intermediate between the levels observed on the lung and brain scaffolds, though the difference in cellularity did not reach statistical significance. In a pilot study, an additional decalcination step in the iWO-DCL protocol resulted in CE bone AOSSs, which were successfully colonized by MDA-MB-231 cells, indicating the relevance to TNBC micrometastases to the bone (Figure A24, Appendix A.4). Our results showed that the ECMs of all tested CE organs are compatible as AOSSs for the 3D culture of MDA-MB-231 cells. As the interactions between lung-specific ECMs obtained by DCL with breast cancer cells were analyzed in recent publications [45,87], in our current study, we focused on detailed analysis of the hepatic micrometastases of TNBC.

#### 4.2.2. Liver ECMs as a Component of the Organ-Specific Metastatic Niche

Liver is a common place of metastatic spread for many types of cancer, including breast cancer [9]. Breast carcinomas are the most frequent primary cancer in young women (20–50 years of age) with liver metastases, and this indicates a pure prognosis [88]. As the role of blood inflow is rather comparable to other carcinomas not associated with the digestive tract, the local tissue-specific mechanisms are hypothesized to be a key factor [18,89] of the high frequency of TNBC metastases to the liver. For instance, special organization of the hepatic vasculature, such as discontinuous basement membranes in sinusoids, allows extravasation of metastatic cancer cells to occur first in the narrow gap between endothelial cells and hepatocytes, known as Disse’s space [90,91,92,93]. It forms a major part of the ECM framework of hepatic parenchyma and contains blood plasma, collagens types I, III, V, VI, and VII, as well as fibronectin and tenascin [94]. Thus, hepatic ECMs (see Figure A4 and Figure A5 in Appendix A) form the homing niche for the incoming cancer cells [90,95,96] and become the operational environment and the regulator of metastatic colonization of the organ [93,97,98].

One of the key observations of the current study was revealed by the existence of iWO-DCL in the parenchymal and stromal compartments with different properties in the CE LS-ECM (see Figure 4). The major molecular components of the CE liver AOSSs were fibrillar collagens, as indicated by Van-Gieson’s and Masson’s trichrome staining (see Figure 4b and Figure 6b), similarly to the liver matrices obtained by perfusion WO-DCL in other species [41,70,71,72,99]. The stromal compartment of LS-ECMs contained more fibrous collagen than the parenchymal one (see Figure 4b), and the architecture of the two compartments was essentially different (see Figure 4a). Considering the ubiquitous presence of collagen in the liver AOSSs and the confirmed preferential attachment of the MDA-MB-231 cells to collagen type I and fibronectin demonstrated by us recently [100], we further concentrated on the analysis of the physical features of the LS-ECM.

Using AFM, we showed that the Young’s modulus of the CE liver scaffolds stored in PBS was ~0.2 kPa (see Figure A6), which is close to the previously reported values for the liver [29]. However, we could not discern the specific mechanical characteristics of the parenchymal and stromal ECMs in hydrated AOSSs due to the extensive surface topography. Therefore, we applied our original methodology [62] and examined the dehydrated and deparaffinated unstained histological sections of the CE liver AOSSs by AFM. This allowed us to reveal that the two liver ECM compartments differ at both the micron and submicron scales in their roughness (the parenchymal ECM is rougher) and at the submicron scale in their stiffness (the stromal ECM is stiffer) (see Figure 5, Figure A7 and Figure A8). The obtained stiffness values (in the GPa range) are considered to be characteristic of collagen fibrils [29].

#### 4.2.3. Colonization of the Liver ECM by TNBC Cells

Adhesion patterns. Upon first contact with the LS-ECM, the TNBC cells preferentially attached to the matrix of the stromal compartment, compared to the almost three times less frequent binding to the parenchymal ECM (see Figure 6a,b). We think that this indicates that the perivascular ECM forms the most attractive niche for TNBC cells, while the initial entrance of the disseminating cancer cells happens via the Disse’s space ECM (parenchymal compartment of the LS-ECM). The higher concentration of collagen (see Figure 4b), stiffness, as well as the relatively larger available space for attachment (decreased roughness means the extended smoother surfaces) of the stromal ECM of the liver may be the probable drivers of the selective higher seeding of TNBC cells in the stromal compartment.

Our findings correspond to the literature, indicating that a number of factors could play a role in the observed differential patterns of cellular adhesion. For example, the spatial gradients [101,102] of ECM components between the triads, central veins, and Disse’s space (as it is illustrated and commented in Figure A4 and Figure A5); the density or stiffness [103,104,105]; the roughness (as observed in the current study); and the porosity or confinement [106,107] of the matrix of hepatic parenchyma and stroma. These properties of ECMs cannot be decoupled in the current model system [108] and require intentional reduction of the complexity of the operational environment of the cells.

A limitation of our study is the unknown precise spatial distribution of the ECM proteins in the scaffolds. This is due to the limited availability of reliable immunohistochemical (IHC) tools that reproducibly work in avian tissues and the intrinsically non-quantitative nature of IHC studies. However, in an earlier work, we confirmed the significance of the ECM protein composition for the MDA-MB-231 cell attachment. On controlled ECM microarrays, we revealed preferential adhesion to collagen type I and fibronectin-containing substrates and moderate-to-low cell attachment to the components of basement membranes [100].

Clustering. During the first two weeks of culturing, TNBC cells in the parenchymal compartment of LS-ECM AOSSs mainly remained individual or formed very small groups (<10 cells). At the same time, the stromal compartment was populated mostly by multicellular clusters. Such clustering may be associated with the response of MDA-MB-231 cells on a notably increase of the stiffness, as we recently reported [100].

Differential invasion and cellular phenotype plasticity as the mechanisms of metastatic colonization. The TNBC cells demonstrated different invasion patterns in the parenchymal and stromal LS-ECM compartments. The invasion in the parenchymal compartment emerged as amoeboid migration [109], as the cells mainly preserved their round epithelial morphology (see Figure 6c) and appeared to be distanced from one another while permeating through small ECM voids without histological signs of matrix degradation. The stromal compartment was initially invaded by the clusters of cancer cells, therefore indicating that collective cell migration [109] was the early mechanism of the colonization in the stromal ECM of the liver. The phenotype of the cells in the stromal compartment was partially skewed toward elongated (mesenchymal-like) ones (see Figure 6c). These alternative colonization strategies resulted in different depths of the invasion fronts in the parenchymal and stromal compartments, found to be ~130 and ~70 µm (see Figure 6a and Figure A10) in week 1, respectively, implying that individual cell migration in the former hepatic parenchyma resulted in a faster speed of the invasion front (~10 µm/day in the current experimental conditions), while the stromal parts of the liver matrix were invaded more slowly (~5 µm/day) by TNBC cellular cohorts. This is consistent with the in vitro data, indicating that individual cancer cell migration is faster than the collective mode due to different adhesion mechanisms and reactions to spatial confinement [110].

From week 3 onward (see Figure 6 M, W4), the TNBC cells invaded the LS-ECM scaffolds to the depth of 800–1500 µm from the surface. This coincided with visible remodeling of the stromal ECM emerged as its “spongification” and gradual blurring of the difference between the liver ECM compartments. The cellular population comprised a mix of cells with epithelioid and mesenchymal-like morphologies. A significant increase in the invasion front speed was observed (to ~26–50 µm/day) during this period.

This can be associated with the simultaneously observed reversible phenotype transitions (from epithelial-to-mesenchymal-like state and vice versa, EMT and MET, respectively) and the matrix remodeling, probably employed by the TNBC cells as colonization strategies. This is in agreement with the reported interdependence between the morphology and the preferred migration mode of the cells, with reversible transitions between the mesenchymal and epithelial phenotypes [111]. The observed changes in the cell shapes that depended on the contact with the parenchymal or stromal ECMs reflect the adaptability of the cells to the inhomogeneous ECM landscape. The epithelioid morphotype dominated in the parenchymal compartment. The mesenchymal-like shapes temporarily emerged mostly in the stromal compartment. Such high phenotypic plasticity of the TNBC cells colonizing different compartments of the LS-ECM, therefore, occurred rather by swaying than switching between the epithelial and mesenchymal-like morphology, EMT and MET. This corresponds well with the concept of partial EMT as a mechanism of cancer cells adaptation to the specific niches [112] and a very important mechanism of the metastatic organotropism [10].

Implications for the treatment and diagnostics of micrometastases. The cellular density in the parenchymal compartment by the end of the second week after seeding remained more than two times lower than in the stromal zones, suggesting that the proliferation and migration states of the cells could be mutually exclusive. A similar phenomenon was observed in malignant gliomas [113]. We conclude that early TNBC colonization of the parenchymal and stromal compartments of the liver ECM occurs via different mechanisms and may require different diagnostic and therapeutic approaches. For example, the treatment of the micrometastases located in the liver parenchyma should be focused on cellular immobilization, and on cytostatic action for the micrometastases in the hepatic stroma. Interestingly, our current findings of the cell clustering in the stromal compartment of the LS-ECM are consistent with the detection of non-vascularized hepatic metastases presented as cellular clumps via Doppler sonography near the liver portal triads [114]. The observed pattern of TNBC cell compartment-specific distribution across the LS-ECM also explains the negligible detectability [17,115,116,117] of single-cell occult metastases in the liver parenchyma vs. the cell clusters near blood vessels and other stromal components. Then, an enhanced detection sensitivity is needed to reveal the micrometastases hidden in the liver parenchyma. Together, these observations show that the development of compartment-specific drug delivery and contrasting agents, for example, targeted nanomedicines, may be an efficient way to control early liver micrometastases of TNBC.

#### 4.2.4. MDA-MB-231 Cell Growth in 2D Monolayer and 3D TEC Cultures in Vitro

Our results showed essentially different cell population dynamics in the 2D culture of MDA-MB-231 cells and in 3D TECs (see Figure 6d). The plastic-cultured cells grew much faster during week 1 than cells in the 3D TECs. Later, in the 2D cultures, the growth rates stabilized and even decreased in week 4, while in 3D TECs the cell viability was steadily increasing during the first three weeks. We found that a logistic growth model was applicable to both cultures. Interestingly, such a model accurately captured the growth of breast carcinoma cells in large clinical datasets [118]. By its nature, the logistic growth mode indicates the presence of resource limitations [119]. In the 2D cultures, this is likely due to the saturated confluence, which was reached on the first week. This was not the case for the TECs, because of the initially smaller numbers of attached cells and the larger total surface area of the porous ECM scaffolds. We attribute the decrease in the cell growth rate in TECs after week 3 to metabolic limitations of 3D cell culture, such as relative hypoxia at the depth of the constructs [120]. This agrees with our histological observations of the secondary necrotic areas forming at the late stages of TECs development. This is additionally supported by the observation of the linear cell growth near the surface (the part with better oxygenation and nutrition) in 3D TECs (see Figure A10f in Appendix A.3.3).

#### 4.2.5. Results of the CAM Assay

We found that the grafted TECs induced an enhanced angiogenic response in comparison to natural embryonic CAM vascularization. In contrast to natural embryonic angiogenesis, which occurs within the studied incubation age mainly by intussusceptive microvascular growth (i.e., splitting of the existing capillaries along the blood vessel axis) [121], the grafted tumors stimulated branching of the host blood vessels, indicating that sprouting angiogenesis was taking place. Interestingly, the increase in branching, in contrast to the increase of blood vessel length density, was not induced by grafted cell suspensions, indicating the contribution of the ECM-related factors to the tumor-specific angiogenic potential of the TECs. The sprouting angiogenesis is the key part of the angiogenic switch in the metastatic progression [7,122]. Taken together, our results indicate that the presented model of early TNBC liver micrometastases is biologically accurate and reflects the transitional avascular state of the metastatic tumor tissue that can induce angiogenic switch and transform into the blood-perfused macrometastases.

### 4.3. Feasibility of 3D Engineered Models of Cancer Micrometastases for the Testing of Drugs and Nanoparticles

Finally, we examined the feasibility of the 3D tissue engineering TNBC liver AOSS model for drug and nanomedicine research. It is considered that the use of nanoscale diagnostic and therapeutic agents is a promising approach to address the challenges of the detection and treatment of micrometastases [117]. We found that the sensitivity of TNBC cells to the chemotherapeutic drug Dox in free and nanoformulated forms was significantly reduced in the 3D liver AOSS-based TECs, in comparison to the 2D cultures, with a 9-times increase in EC_50_ values and a value of over 10 µg/mL for IC_50_. This agrees with earlier reports that additional signaling and diffusion inputs from the 3D microenvironment and ECM lead to decreased responses to chemotherapeutics [123]. Another factor contributing to the reduced response on the cytostatic treatment may be the difference in the growth rates between 2D and 3D model systems, corresponding with the stronger cytostatic effect of Dox in the faster proliferating cells in 2D. We also observed a trend of a higher therapeutic effectiveness of the nanoformulated Dox in 3D TECs in comparison to free Dox, notable at high doses of doxorubicin (Figure A21). This result was corroborated by fluorescence imaging, where an uptake and intracellular distribution of free and nanoformulated Dox were found to be comparable in the 2D TNBC cultures, while not in the 3D TECs. In the TECs, the Dox released from AMS-6-Dox nanoparticles and almost completely killed deeply located cells. In contrast, free Dox accumulated not only in the cell nuclei, but also notably accumulated in the ECMs (Figure 6b). We tentatively attribute this difference to the faster drug release from AMS-6-Dox (see Figure A15c in Appendix A.3.5) and electrochemically affected transport of the negatively charged AMS-6-Dox (see Table A5 in Appendix A.3.5.1) across the net-negatively charged LS-ECM. This observation suggests that the potential reduction of the cytotoxic side effects of free Dox can be realized by appropriate nanoscale delivery agents able to prevent accumulation of this drug in liver ECM. However, the detailed mechanisms of the observed phenomena require further studies.

## 5. Conclusions

In conclusion, we demonstrated a novel engineered 3D micrometastatic cancer model that fills the gap between conventional cell cultures and in vivo testing in animals. It provides an affordable and facile platform for drug and nanomedicine development and cancer research. This model allowed us to reveal distinct, stage-specific, and previously unknown ways of metastatic colonization of the liver by TNBC cells and contributes to the understanding of the mechanisms of metastatic organotropism. The compartmentalization of the liver ECM into dual parenchymal vs. stromal niches was found to be the key determinant of the colonization pattern used by TNBC cells. Cells cultured in a 3D liver-derived matrix demonstrated increased resilience to free and nanoformulated doxorubicin. The biological relevance of the model was confirmed in vivo by induction of an angiogenic switch in the host tissue by the grafted TECs. As decellularized tissues have negligible cross-species differences and can be used as xenotransplants, this methodology is highly universal and may be applied to other types of chick embryo organs and other cell lines and tumor types.

## 6. Patents

The iWO-DCL procedure has been temporarily protected by an Australian provisional patent, no. 2018903766, by A. Guller, A. Nadort, and E. Goldys (lapsed).

## Data Availability

The relevant data generated and (or) analyzed in the current study are available from the corresponding author upon reasonable request.

## Supplementary Materials

The following are available online at https://www.mdpi.com/article/10.3390/biomedicines9111578/s1.

## Appendix A

### Appendix A.1. Design of the Study on the Feasibility of the 3D Tissue Engineered Model of TNBC Micrometastases to the Liver

The LS-ECM -TNBC 3D model design and applications are schematically illustrated in Figure A1. As a source of donor LS-ECMs, we used chick embryos incubated until embryonic day 18 (ED18), when all major organs had formed, before pain sensitivity had fully developed [124]. After the extraction of the chick embryo livers, the organs underwent iWO-DCL and then were cut by a 4 mm biopsy punch to obtain acellular organ-specific scaffolds (AOSSs). Each individual AOSS presented an 8–10 mm^3^/2–3 mm side size fragment of decellularized chick embryo livers allowing to preserve histoarchitectural features of the LS-ECMs. Next, 3D TECs were created by seeding of MDA-MB-231 cells on the top of AOSSs placed in the individual wells of the culture plate. The cells grew on and within the scaffolds in a static in vitro culture for up to four weeks, allowing us to characterize the cell invasion patterns, examine drug responses, and validate in vivo the cancer-specific behavior after implantation in chick embryos. The TECs were sampled for histological and other analyses at various time points during the in vitro culture (up to four weeks after cell seeding). Matching 2D in vitro cultures of the same cells were studied in parallel with the TECs. The TECs with well-developed cell colonies (after three weeks of in vitro culturing) were used for evaluation of cytotoxicity and cellular uptake of doxorubicin (Dox), as well as Dox-loaded mesoporous silica nanoparticles (Anionic Mesoporous Silica-6, AMS-6-Dox) [125]. The ability of the engineered tumors to induce an “angiogenic switch,” or growth of blood vessels in the host tissue, was examined by chick embryo chorioallantoic membrane (CAM) assay via grafting of the TECs [126]. This effect is known to be a critical cancer hallmark [127], especially significant for metastatic progression [5,6,7].

### Appendix A.2. Decellularization of Multiple CE Organs

During the preliminary experiments, four protocols of iWO-DCL were tested by macroscopic and histological examination of the tissues, including 1% SDS, 0.1% SDS, 1% Triton-X-100, and a sequential combination of the Triton-X-100 and SDS 0.1%. As a result, we found that the maximal degree of the organ-specific ECM preservation and the most efficient removal of cellular debris were achieved by the application of 0.1% SDS. An overview of the macroscopic changes of various ED18 CE organs during the iWO-DCL is shown in Figure A1. The organ-specific iWO-DCL timelines are presented in Table A1.

**Table A1:** Recommended protocols of iWO-DCL of ED18 chick embryo organs and tissues.

Organs	Average DCL Time, h	Minimal Washing Time, h	Recommended Shaking Speed during DCL^3^, rpm
Brain ^1^	~48	48	100 (first 24 h), 90 (second 24 h)
Heart	~180–200	~48–60	150 (first 24 h), 120 (second 24 h)
Lungs	~48	48	100 (first 24 h), 90 (second 24 h)
Liver	~240–280	48	120 (first 5–6 days), 50 (the rest of the time)
SI ^2^	~48	48	150 (first 24 h), 120 (second 24 h)
PV ^2^	~90–120	48	150 (first 24 h), 120 (the rest of the time)
Ve ^2^	~250–280	~48–60	150 (first 24 h), 120 (the rest of the time)
Spleen	~36–48	48	150 (first 24 h), 120 (the rest of the time)
BMu^2^	~90–120	48	150 (first 24 h), 120 (the rest of the time)
Skin	~90–120	48	150 (first 24 h), 120 (the rest of the time)

^1^ The fragmented material may be collected by centrifugation at 10,000 rpm × 10 min. ^2^ Abbreviations: SI—small intestine; PV—proventriculus; Ve—ventriculus; BMu—breast muscle. ^3^ The shaking speed has to be adjusted depending on the shaker model (radius of the orbit) in order to avoid mechanical damage of the tissues. This may also depend on the volume of the organs or tissues vs. the volume of the vessel. The recommended speed is validated with the ratio of the tissues/total volume ratio, such as approximately 1:7 (e.g., 5 mL of tissues in 35 mL of DCL/washing solution per 50 mL Falcon tube). Note that the vessel should have an empty volume (in the current illustrative case it is 15–20 mL) to allow free movement of the contents.

### Appendix A.3. Development and Feasibility Testing of 3D Tissue Engineering Model of TNBC Micrometastases to the Liver

#### Appendix A.3.1. Native Structure of Chick Embryo Liver Tissue and the Effect of Decellularization

The chick embryo livers investigated in this work on ED18 had a typical hepatic histoarchitecture with vascular and biliary elements, which, however, appeared a bit less distinctive than in normal human liver tissue (Figure A1a). The stromal elements of the liver tissue, including central veins and portal triads, contained slightly more fibrous collagen than the hepatic parenchyma (Figure A1b), while the capsular and subcapsular connective tissue structures (Figure A1d) had significant local concentration of mature fibrous collagen. The tissue was completely orthochromatic when stained by toluidine blue (Figure A1c).

#### Appendix A.3.2. Effect of iWO-CL on CE Livers

The iWO-DCL procedure preserved the shape of the liver, but its volume decreased significantly and the decellularized organ became almost translucent (Figure A2). The cells and cellular debris were no longer observable in the DCL tissue by histological methods (Figure A3). The hepatic parenchyma transformed into a loose, fine, mesh-like matrix formed mainly by the residuals of perisinusoidal ECM of Disse’s spaces (Figure A3). The former septal connective tissue and portal elements (portal arteries and veins) and former central veins were identified in the DCL organ by their denser ECM and morphology in hematoxylin and eosin-stained sections (see Figure A3a) and by a slightly higher collagen concentration (pink staining by the Van Gieson’s method; see Figure A3b). After the IWO-DCL, two compartments of the LS-ECM became distinguishable. They are further referred to as the “parenchymal” compartment, comprising a mesh-like matrix of the former parenchyma, and the “stromal” compartment, comprising the DCL portal vasculature, interlobular septal stroma, and acellular walls of former blood vessels, including central veins and portal arteries and veins (Figure A3). The effect of iWO-DCL on the separate histological elements of the liver tissue is schematically presented in Figure A4. The location of the Disse’s space and the composition of the ECM of the Disse’s space, according to the literature data [94,101,102,128], are shown in Figure A5.

#### Appendix A.3.3. Structural and Morphometrical Analysis of 3D TECs

**Table A2:** Relative cellularity (mean% ± standard deviation of the TEC section area) in different compartments of the liver ECM measured as a ratio of cells to matrix area: Results of image analysis of histological sections. Ten images were analyzed for each timepoint/compartment.

Sampling Time Point	Compartment		
	Parenchymal	Stromal	Mixed
**Week 1**	5.1 (±6.9)	16.3 (±15.2)	-
**Week 2**	6.8 (±6.5)	17.3 (±9.4)	5.5 (±3.4)
**Week 3**	10.0 (±3.0)	37.7 (±21.0)	10.6 (±7.8)
**Week 4**	1.8 (±1.4)	1.2 (±NA)	5.6 (±NA)

**Table A3:** Estimated values of the logistic growth model parameters with confidence intervals (CI_95%_).

Parameters	2D		3D	
	Estimated Value	CI_95%_	Estimated Value	CI_95%_
**C_0_**	0.03181	−0.2501, 0.3137	0.006906	−0.01293, 0.02675
**C_max_**	0.8891	0.6392, 1.139	0.1524	0.1107, 0.1941
**d**	0.6325	−0.7616, 2.027	0.2826	−0.005646, 0.5708

**Table A4:** The goodness of fit of the logistic growth model.

Characteristics of Fitting	2D	3D
**SSE**	0.01974	0.0002222
***R*-squared**	0.9634	0.9864
**Adjusted *R*-squared**	0.9267	0.9729
**RMSE**	0.09935	0.01054

#### Appendix A.3.4. Angiogenic Assay on Chick Embryo CAMs

##### Appendix A.3.4.1. Egg Preparation and Grafting Procedure for Angiogenic Analysis

The experiment was approved by the Animal Ethics Committee of Macquarie University (ARA 2015/006). The fertilized White Leghorn chicken eggs were incubated for 72 h, until embryonic day 3 (ED3). Then, the eggs were placed in a horizontal position and left in the incubator for the next 30 min for repositioning of the embryos. Afterward, the tops of the blunt ends of the shells were wiped with 70% ethanol, and 3–4 mL of the egg white was extracted from the bottom part of the egg by puncturing of the blunt end of the shell with a syringe needle (18G) at the angle of ~45° (Figure A11, day 3). The albumin extraction resulted in a decrease in the total volume of the egg and dropping of the chorioallantoic membrane (CAM), which was necessary for the further grafting procedure. After the extraction, the stab holes were sealed with a sticky tape and the eggs were returned to the incubator until ED8. On ED8, the eggshells were cut on the blunt end to create a lid and expose the CAM. Following this, the grafting procedure was performed (Figure A11, day 8).

All of the embryos were divided into four groups, with 10 eggs per group. Group 1 was used as a control (labeled “Control”) and left ungrafted in order to evaluate the parameters of natural angiogenesis that occurred in chick embryos during the period between ED8 and ED12. In the other groups, the following materials were aseptically implanted onto the CAM under sterile conditions: (1) Chick embryo liver AOSSs soaked in CCM following the pre-seeding protocol (labeled “scaffold”) for 24 h; (2) cell suspension of MDA-MB-231 cells, 2 × 105 cells in 60 µL of CCM (labeled “cells”); and (3) the TECs, prepared as described above and cultured in vitro for 12 days prior to grafting (labeled “TECs”). This time period of preliminary culture of TECs in vitro was chosen to obtain the engineered tumor samples containing the similar number of cells (~ 2 × 10^5^ cells per sample) in the compared TECs and cell suspension xenografts. This period was also preferred to obtain the TECs with actively grown cell populations.

Following this, the images of the CAM were taken with a stereomicroscope as described in Section Appendix A.3.4.2 below. Then, the eggs were sealed and returned back to the incubator and maintained under standard conditions with excluded rotation. Afterward, on ED12, the eggshells were re-opened, and the imaging was repeated under the same conditions as on ED8. After the imaging session on ED12, the chick embryos were euthanized by quick decapitation. The CAM with grafter materials and control samples of ungrafted CAM were dissected, washed in PBS, and studied by histological methods.

##### Appendix A.3.4.2. Imaging of CAM In Vivo

The CAMs of the eggs with open shell lids were imaged in vivo using the Olympus MVX10 (Olympus, Japan) stereomicroscope equipped with an eyepiece magnification ranging between 0.63× and 6.3×, a fixed focus, and two objective lenses, 1×/0.25 N.A. and 2×/0.5 N.A. The microscope featured a long working distance (20–87 mm), adjustable field-of-view (FOV; 1.7–55 mm in diameter). To maintain the healthy state of the chick embryos, the heating stage ThermoPlate by Tokai Hit (Shizuoka-ken, Japan) was used during in vivo inspection of the CAM. The objective 1×/0.25 N.A. was applied for the rough focusing and the lens magnification of 2× was used for the detailed imaging. The samples were illuminated from above by a 100 W mercury lamp. In order to achieve a higher contrast of the red color of the blood, a filter cube with blue illumination light was used. This filter cube contained one single-edge short pass dichroic beam splitter (Semrock, USA) with transmission of the wavelengths above 750 nm and a single-band bandpass (510,570 nm) filter as the emission filter (Semrock, USA). The microscope was coupled with a motorized Z focus ProZ Stand (Prior Scientific, USA), which provided seamless zooming from 40× to 1250× magnification (used for focusing and study purposes).

##### Appendix A.3.4.3. Quantification of Angiogenesis

We evaluated the morphological parameters of angiogenesis by determining the quantitative features of newly developed vessels within the CAMs on ED8 and ED12. We analyzed images of five eggs for each experimental group (intact eggs, cell suspensions, scaffolds, and TECs). In particular, we quantified the density (number per unit area) and length of major blood vessel sections between the branching points (segments), the blood vessels of the first order (extremities) [129], and the density of branching points [64] (Figure A12). In this approach, a branch from one major vessel creates two new vessels; also, the end of a vessel is counted as a branching point. Other angiogenesis parameters are calculated as described elsewhere [64,129]

The main steps of the image analysis algorithm are the subtraction of the background of the image, the adaptive thresholding, noise reduction, and the skeletonization, as described in References [129,130] and . To reduce the noise, erosion and dilation transformation were applied individually on each image.

After skeletonizing, the ImageJ plugin Angiogenesis Analyzer [66] was applied to each egg and four ROIs were selected, representing four opposite sides of the graft. The plugin estimates the number of branching points and total vessel length, whereas the vessels are divided into different elements. The detailed description and illustration of terminology used for quantification of binary tree of blood vessels structure can be found in the ImageJ documentation [66]. Statistical significance of the differences between the studied groups’ parameters of blood vessel trees was analyzed by a one-sample Kruskal–Wallace test, followed by comparisons in the pairs of the groups with use of the Mann–Whitney *U*-test.

The results (Figure A13) show a statistically significant increase in the following parameters per unit area in TECs on ED12 in comparison to intact controls (normal embryonic angiogenesis, ED12): The total blood vessel length or vascular density (*p* = 0.07) (Figure A13a); the number of junctions (*p* = 0.020) (Figure A13b); the length of segments (the parts of the blood vessel tree between two enclosed neighbor branching points) (*p* = 0.003) (Figure A13c); the number of first order vessels (extremities) (*p* = 0.014) (Figure A13d); the number of branches (*p* = 0.039) (Figure A13e); and a statistically non-significant increase in the length of branches (*p* = 0.121). Statistically significant increase of the number of branching points per area (*p* = 0.020) is illustrated in the main text of the paper (see Figure 8g). Unseeded scaffolds and cancer cell suspensions were found not to affect normal embryonic angiogenesis, as there were no statistically significant differences between the scaffolds and intact controls.

Moreover, there were no statistically significant differences in the parameters of the blood vessel tree architecture measured on ED8 between the studied groups (data not shown).

#### Appendix A.3.5. 3D TECs of TNBC Micrometastases to the Liver as Drug and Nanomedicine Testing Platform

##### Appendix A.3.5.1. Characterization of Nanoparticles

**Table A5:** Zetapotential of AMS-6 and AMS-6-Dox nanoparticles.

Sample	Temperature, °C	pH	Polydispersity Index ((Dw/Dm)^2^)	Zeta-Potential, mV
Pure AMS-6 in PBS	25	7.2	0.544	−10.9
Pure AMS-6 in CCM	25	7.4	0.685	−8.3
AMS-6-Dox in PBS	25	7.2	1.000	−11.2
AMS-6-Dox in CCM	25	7.4	1.000	−8.8

##### Appendix A.3.5.2. Effect of Free and Nanoformulated Doxorubicin on MDA-MB-231 Cells in 2D and 3D (TECs) Cultures

**Table A6:** The results of fitting a dose–response model to the measurements of cell viability. Estimated values of the dose–response model parameters for free Dox in 2D and 3D.

	2D		3D	
	Estimated Value	Standard Error	Estimated Value	Standard Error
Maximum effect, %	64.85	2.83	34.34	2.71
EC_50_, µg/mL	0.27	0.09	2.40	0.41
Hill coefficient	3.20	1.27	0.68	0.39
IC_50_, µg/mL	0.43	0.12	>10	

**Table A7:** The results of fitting the dose–response model to the measurements of cell viability. Estimated values of the dose–response model parameters for AMS-6-Dox in 2D and 3D.

	2D		3D	
	Estimated Value	Standard Error	Estimated Value	Standard Error
Maximum effect, %	63.06	1.97	40.53	5.47
EC_50_, µg/mL	0.26	0.06	2.38	0.86
Hill coefficient	2.81	0.80	0.34	0.18
IC_50_, µg/mL	0.46	0.10	>10	
